# In‐Depth Cell‐Type‐Specific Proteome Landscape of the Brain from Human Amyloid‐β Overexpression Mouse Model

**DOI:** 10.1002/advs.202409318

**Published:** 2025-05-08

**Authors:** Taekyung Ryu, Seok‐Young Kim, Thujitha Thuraisamy, Jisu Shin, Yura Jang, Tae‐In Kam, Chan Hyun Na

**Affiliations:** ^1^ Department of Neurology Johns Hopkins University School of Medicine Baltimore MD 21205 USA; ^2^ Neuroregeneration and Stem Cell Programs Institute for Cell Engineering Johns Hopkins University School of Medicine Baltimore MD 21205 USA; ^3^ Department of Brain and Cognitive Sciences Korea Advanced Institute of Science and Technology Daejeon 34141 South Korea

**Keywords:** biotin‐tyramides, brains, cell‐type‐specific proteomes, iCAB, biotinylation, immunohistochemistry, mass spectrometry

## Abstract

Amyloid‐β (Aβ) plays a crucial role in Alzheimer's disease pathogenesis. Understanding how Aβ overexpression alters the proteome of individual brain cell types is essential but challenging due to the nature of brain tissue, which contains intermingled various cell types. The current methods for cell‐type‐specific proteomics either require genetic modifications or complex cell isolation, limiting their use. This study introduces a novel method, in situ cell‐type‐specific proteome analysis using antibody‐mediated biotinylation (iCAB), which applies immunohistochemistry with biotin‐tyramide to target cell‐specific proteins directly in tissue. Applied to 5xFAD mice, iCAB enables us to identify ≈8000 cell‐type‐specific proteomes with significantly more differentially expressed proteins than traditional bulk proteome methods, pinpointing unique pathways such as mRNA processing, calcium regulation, and phagocytosis for neurons, astrocytes, and microglia, respectively. This study reports in‐depth the cell‐type‐specific brain proteome landscape of the human Aβ overexpression mouse model for the first time using an innovative tool that is powerful, straightforward, and applicable to both animal models and human tissues, without the need for prior genetic alterations.

## Graphical abstract

In situ Cell‐type‐specific proteome Analysis using antibody‐mediated Biotinylation (iCAB) is a method that directly targets and analyzes proteins in specific cell types within tissue. Applied to 5xFAD mice, iCAB identifies ≈8000 cell‐type‐specific proteins, revealing unique pathways in neurons, astrocytes, and microglia. This technique offers a powerful and straightforward approach applicable to both animal models and human tissues.

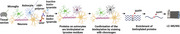

## 1 Introduction

Amyloid‐β (Aβ) plays a pivotal role in the pathogenesis of Alzheimer's disease (AD), acting as a cornerstone in the complex cascade leading to neurodegeneration.^[^ ^1^ ^]^ Accumulation of Aβ in the brain, forming insoluble plaques, is a hallmark of AD and is believed to initiate a sequence of events that lead to synaptic dysfunction, neuronal loss, and cognitive decline characteristic of the disease.^[^ ^2^ ^]^ This amyloid cascade hypothesis is supported by genetic, pathological, and biochemical studies, demonstrating that mutations increasing Aβ production are linked with familial forms of AD and that Aβ accumulation precedes and predicts tau pathology and neurodegeneration.^[^ ^1^, ^3^ ^]^ However, the pathogenesis mechanism mediated by Aβ has not been elucidated yet.

Proteins are essential players and functional operatives within cells.^[^ ^4^ ^]^ Therefore, examining proteome changes in the cell is essential to understanding both normal physiological processes and the development of various diseases. While tissues of higher organisms, like humans and animals, are composed of multiple cell types intercalated with each other, the conventional mass spectrometry‐based proteomics method has been limited to reading the sum of their changes, compounding the interpretation of intricate proteome changes that occurred by multiple cell types.^[^ ^5^ ^]^ To overcome these limitations, various attempts have been made to analyze cell‐type‐specific proteomes. The bioorthogonal noncanonical amino acid tagging (BONCAT) method employs the cell‐type‐specific expression of a mutant type of methionyl‐tRNA synthetase (MetRS*) that recognizes a noncanonical amino acid, azidonorleucine (ANL). Thereby, ANL can be incorporated into the cell type where MetRS* is expressed. The ANL‐incorporated proteins could be biotinylated by click‐chemistry.^[^ ^6^ ^]^ Methods such as enhanced ascorbate peroxidase 2 (APEX2) and in situ cell‐surface proteome extraction by extracellular labeling (iPEEL) use cell‐type‐specific peroxidases expressed in subcellular organelles or on the cell surface in the mouse brain. These peroxidases can then biotinylate proteins in their vicinity.^[^ ^7^, ^8^ ^]^ Similarly, the TurboID method employs the cell‐type‐specific expression of TurboID in the mouse brain, which can biotinylate nearby proteins.^[^ ^9^ ^]^ The biotinylated proteins in these approaches could be enriched by avidin‐based enrichment and identified by mass spectrometry. All these approaches require the cell‐type‐specific expression of an enzyme through transgenic or gene‐targeting approaches. As such, they are not readily adaptable to broad applications. Moreover, these approaches have limited proteomic depth as the number of identified proteins was limited to <3500. Beyond the chemical tagging approaches, physical separation techniques like laser capture microdissection (LCM)^[^ ^10^ ^]^ and fluorescence‐activated cell sorting (FACS)^[^ ^11^ ^]^ have also been utilized. However, the LCM method is tedious, time‐consuming, and often needs expertise in anatomy and histology. On the other hand, FACS involves dissociating the target cells, which can lead to a significant loss of proteomic information that exists in vivo.^[^ ^12^ ^]^

**Figure 1:**
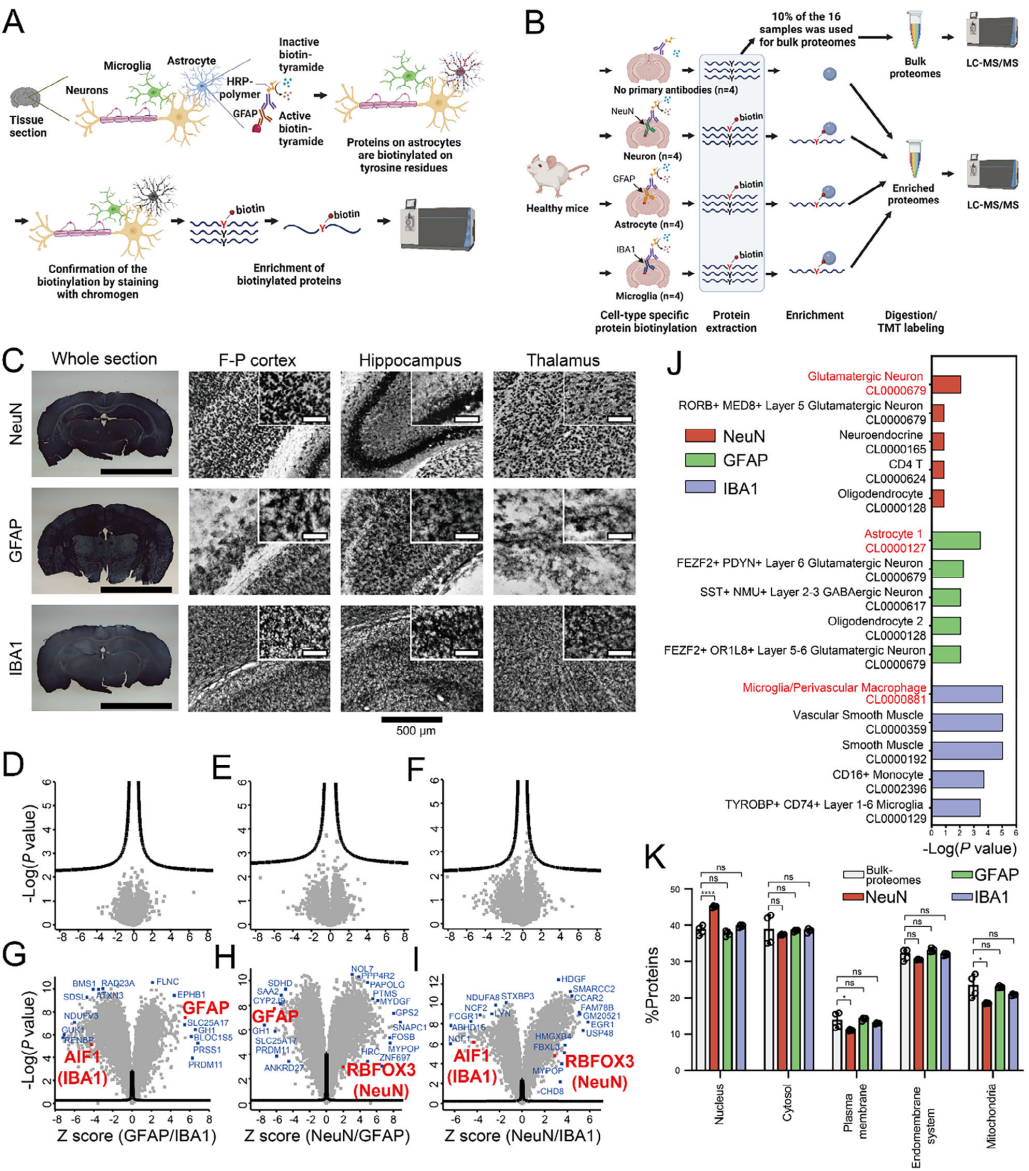
Development of an in situ cell‐type‐specific proteome analysis of the brain using antibody‐mediated protein biotinylation. The concept of iCAB for the cell‐type‐specific proteomic analysis (A). Horseradish peroxidase (HRP)‐conjugated to a secondary antibody can be localized to a specific cell type (e.g., astrocyte) through IHC targeting cell‐type‐specific marker protein (e.g., GFAP). HRP will then activate biotin‐tyramide, and, in turn, the activated biotin‐tyramide will biotinylate proteins of the target cell types. The biotinylation can be confirmed using an ABC kit. The biotinylated proteins can be enriched using streptavidin‐sepharose beads and released by on‐bead digestion. The resulting peptides can be identified by LC‐MS/MS. Schematic diagram of iCAB study using mouse brain sections (B). A proof of principle experiment for iCAB was conducted using anti‐NeuN (NeuN), anti‐GFAP (GFAP), and anti‐IBA1 (IBA1) antibodies to target neurons, astrocytes, and microglia, respectively, in healthy mouse brains. A negative control, in which no primary antibody was treated, was also included to estimate the non‐specific biotinylation. The biotinylated proteins were enriched, followed by on‐bead digestion. The digested peptides were labeled with 16‐plex TMT, pooled, and prefractionated using basic pH RPLC. The fractionated peptides were analyzed by LC‐MS/MS. Ten percent of the iCAB‐processed mouse brain lysates were subjected to direct proteome analysis without enrichment for biotinylated proteins to compare the bulk proteome with cell‐type‐specific proteome. Chromogen‐stained images of the biotinylated neurons, astrocytes, and microglia in the frontoparietal (F‐P) cortex, hippocampus, and thalamus regions (C). The insets in the F‐P cortex, hippocampus, and thalamus regions are for magnified images, where the scale bar length is 100 µm. The scale bar length at the bottom of the whole section images is 5 mm. The three volcano plots at the top (D–F) represent the bulk proteome data (*n* = 4, technical replicates for each group), which were processed for iCAB but not enriched. The three volcano plots at the bottom (G–I) represent cell‐type‐specific proteome data (*n* = 4, technical replicates for each group), which were processed for iCAB and enriched. Proteins located outside the curved lines have *q*‐values less than 0.05, indicating significant differential expression. Data were transformed using z‐scores. *P* values between comparison groups were calculated using Student's two‐sample (unpaired, two‐tailed) *t*‐test, and *q*‐values were calculated using significance analysis of microarrays (SAM), with permutation‐based FDR estimation and an *S*₀ value of 0.1. Plots of Azimuth cell type analysis using the top 300 differentially expressed proteins for each cell type with the lowest *q*‐values (J). Red, green, and purple squares represent the results for neurons (NeuN), astrocytes (GFAP), and microglia (IBA1). The top five cell types identified in each cell type proteome data were listed with ‐log (*P* value) in this plot. The most dominant cell types were indicated with red font. Subcellular distribution of iCAB‐enriched proteome in each cell type (*n* = 4, technical replicates for each group) by Gene Ontology Cellular Compartment (GO‐CC) annotations (K). This GO‐CC data represents the ratio of identified proteins in the bulk proteome, NeuN, GFAP, and IBA1 that belong to the corresponding subcellular compartment. GOCC analyses were performed using all proteins identified by three or more peptides. Data are presented as mean ± standard deviation (SD). An unpaired Student's *t*‐test was used for the statistical analysis (*p < 0.05, ****p < 0.0001, and ns: not significant).

To overcome these limitations, we have developed a novel method that does not require genetic tools, which is called in situ cell‐type‐specific proteome analysis using antibody‐mediated biotinylation (iCAB). This powerful method employs horseradish peroxidase (HRP)‐coupled to a secondary antibody that is localized to a target cell type mediated by the primary antibody specific for a marker protein (e.g., NeuN, GFAP, or IBA1) of the cell type on a tissue section. Thereby, the proteins in the vicinity of HRP are biotinylated in the presence of biotin‐tyramide and hydrogen peroxide. These biotinylated proteins are then enriched using avidin beads and identified through mass spectrometry (**Figure** **1**A). Recently, this in situ antibody‐based biotinylation method was also used for in situ interactome analysis, proving the broad applicability of this antibody‐based biotinylation.^[^ ^13^ ^]^ As a proof‐of‐concept experiment, we employed the iCAB method for neurons, astrocytes, and microglia from mouse brains using anti‐NeuN, anti‐GFAP, and anti‐IBA1 antibodies, respectively. Subsequently, we applied iCAB to 5xFAD and control mice for the cell‐type‐specific proteomic profiling of neurons, astrocytes, and microglia, revealing a deep landscape of cell‐type‐specific proteome changes in the 5xFAD mouse brain. This study provides a novel approach to cell‐type‐specific proteome analysis using antibody‐mediated biotinylation, enabling cell‐type‐specific profiling without the need for transgenic or gene‐targeting strategies. This method is straightforward and user‐friendly, as it can be employed in any research laboratory where immunohistochemistry (IHC) is available. Additionally, it is applicable to any tissue sections, including human tissues, for which IHC is feasible. Therefore, iCAB will open a new avenue to the broad use of cell‐type‐specific proteome analysis, bringing it to the level of routine practice.

## 2 Results

### 2.1 Development of the In Situ Cell‐Type‐Specific Biotinylation Method

To develop cell‐type‐specific protein biotinylation in our study, we first determined the ideal concentrations for primary antibodies. We tested various dilutions (1:100, 1:250, 1:500, 1:1,000) of anti‐NeuN, anti‐GFAP, and anti‐IBA1 antibodies, targeting neurons, astrocytes, and microglia, respectively. Optimal staining contrasts were achieved at dilutions of 1:500 for both anti‐NeuN and anti‐IBA1 antibodies, and 1:100 for anti‐GFAP antibody (Figure S1, Supporting Information), which we used in subsequent experiments. To achieve the maximal biotinylation on the target cell type with minimal biotinylation on the background, the optimal biotinylation condition using biotin‐tyramide is also critical. To ascertain the most efficient biotinylation, we tested various concentrations (0, 0.3, 3, 30, and 150 µm) of biotin‐tyramide. The level of biotinylation on tissue sections was visualized by chromogen after incubating the biotinylated tissue sections with an avidin/biotin‐HRP complex (ABC) kit. As expected, the brain tissue section without biotin‐tyramide did not show any staining. In contrast, other brain tissue sections with biotin‐tyramide showed chromogen staining in a biotin‐tyramide concentration‐dependent manner, supporting that the staining was biotinylation‐dependent (Figure S2, Supporting Information). Though the 150 µm concentration resulted in the darkest staining, the 3 µm concentration provided the best staining contrast across all three cell types. Therefore, we opted to use 3 µm of biotin‐tyramide for the biotinylation process in all three cell types.

We next conducted a proteomic analysis using the cell‐type‐specifically biotinylated tissue sections. To identify proteins biotinylated in a cell‐type‐specific manner, we prepared 40 tissue sections for each of four groups: the NeuN group (anti‐NeuN antibody), the GFAP group (anti‐GFAP antibody), the IBA1 group (anti‐IBA1 antibody), and the control group without primary antibody (Figure 1B). Each group had four replicates, with one out of four replicates per group stained with chromogen to confirm proper biotinylation. The cell‐type‐specific protein biotinylation by iCAB using anti‐NeuN, anti‐GFAP, and anti‐IBA1 antibodies showed distinct cell‐type‐specific shapes indicative of neuronal cell bodies, astrocytes, and microglia, respectively (Figure 1C). The cell‐type‐specific protein biotinylation by iCAB using anti‐NeuN, anti‐GFAP, and anti‐IBA1 antibodies showed distinct cell‐type‐specific shapes indicative of neurons, astrocytes, and microglia, respectively (Figure 1C). The tissue sections were then lysed and de‐crosslinked for paraformaldehyde (PFA)‐linked proteins, followed by enriching biotinylated proteins. Subsequently, they underwent on‐bead digestion and tandem mass tags (TMT) labeling for proteomic analysis. To compare the enriched proteome to the bulk proteome, 10% of the samples from 16 samples were aliquoted before enrichment, followed by being digested, TMT‐labeled, and analyzed by LC‐MS/MS analysis (Figure 1B). We identified 43 059 peptides and 6213 proteins (5966 proteins with quantity) from the bulk proteomics samples and 67 956 peptides and 8430 proteins (8214 proteins with quantity) from the enriched samples. Principal component analysis (PCA) plots revealed that while the bulk proteomics samples showed no distinct clustering, enriched samples displayed clear clustering for each cell type group (Figure S3, Supporting Information). Heatmap analysis further confirmed distinct clustering for each group in enriched samples compared to the bulk proteomics samples (Figure S4, Supporting Information). Notably, chromogen‐stained samples showed no significant difference from non‐stained ones, indicating that both stained and non‐stained sections are suitable for downstream proteome analysis. These results demonstrate successful and distinct enrichments for the four groups.

Subsequently, we conducted statistical analysis for both the bulk and cell‐type‐specific proteomics data to estimate the enrichment efficiency and identify the cell‐type‐specifically abundant proteins. The bulk proteomics data showed no differentially expressed proteins in any comparisons between groups. On the other hand, strikingly, almost all proteins from the cell‐type‐specific proteomics data exhibited differential expressions (Figure 1D–I, Tables S1 and S2, Supporting Information). When comparing the GFAP group with the IBA1 group, we identified 3798 and 3907 up‐ and down‐regulated proteins in the GFAP group, respectively. When comparing the NeuN group with the GFAP group, we identified 3946 and 3863 up‐ and down‐regulated proteins in the NeuN group, respectively. When comparing the NeuN group with the IBA1 group, we identified 2898 and 4771 up‐ and down‐regulated proteins in the NeuN, respectively. Notably, the cell‐type marker proteins such as GFAP, AIF1 (IBA1), and RBFOX3 (NeuN) exhibited differential expressions (Figure 1J). However, when the NeuN group was compared to the GFAP group, the Z score for RBFOX3 (NeuN) was 2, while the proteins with a maximal Z score showed a Z score of >8. This marginal enrichment of RBFOX3 can be explained by the close entanglement of astrocytes with neurons.^[^ ^14^ ^]^ We observed similar findings when one cell type (e.g., neurons) was compared to the two other cell types (e.g., astrocytes and microglia) (Figure S5, Tables S3 and S4, Supporting Information). Additionally, the level of protein identification by endogenously biotinylated proteins or non‐specific binding to streptavidin beads was evaluated by comparing each cell‐type‐specifically enrichment group with the control group that was not treated with antibodies. Remarkably, the abundances of almost all proteins were increased in the NeuN, GFAP, and IBA1 groups compared to the control group, supporting that the iCAB method efficiently enriched the cell‐type‐specific proteins (Figure S6, Tables S5 and S6, Supporting Information). Although most proteins appear to be enriched in a cell‐type‐specific manner, the increased abundance in the enriched proteome compared to the negative control does not necessarily indicate the absence of non‐specifically bound proteins. To assess the proportion of the non‐specifically bound proteins, we conducted the label‐free proteome analysis using a portion of the peptides released by on‐bead digestion. Additionally, we eluted the biotinylated peptides that remained bound to streptavidin after the on‐bead digestion, followed by analyzing them by mass spectrometry. We could identify ≈4500 proteins from the cell‐type‐specific enrichment experiment, while 376 proteins were identified from the negative control, in which no antibody was used. Among these, 326 proteins were consistently identified across all three cell‐type‐specific enrichment proteomes, suggesting that they are non‐specifically bound proteins. On the other hand, no biotinylated peptides were identified in the negative control, further supporting that the proteins detected in the negative control resulted from non‐specific binding. Based on these data, we estimate that 7–9% of the identified proteins may be attributed to non‐specific binding (Figure S7, Supporting Information).

Next, we investigated whether the cell‐type‐specifically upregulated proteins represent the respective cell type. We first selected the top 300 proteins that showed the lowest *q*‐values among the upregulated proteins in each cell type compared to two others and analyzed them using the Azimuth cell‐type annotation reference database in Enrichr. In alignment with our expectations, the top 300 proteins upregulated in neurons, astrocytes, and microglia showed the biggest enrichment toward Glutamatergic neurons, Astrocyte1, and Microglia/Perivascular Macrophage, respectively (Figure 1J, Table S7 Supporting Information). This result demonstrates that the iCAB successfully enriched proteins from respective cell types.

Next, we investigated whether the iCAB method effectively biotinylated proteins across the entire cellular compartments without any bias toward a particular compartment. For this, we performed Gene Ontology analysis using cellular compartment terms for both the bulk and cell‐type‐specific proteomics data. Both the bulk and cell‐type‐specific proteomics data displayed a similar distribution across subcellular compartments like the nucleus, cytosol, and mitochondria (Figure 1K). One noticeable difference between the bulk and cell‐type‐specific proteomics data is that NeuN showed a slightly higher abundance for the nucleus proteins. We consider that this is because NeuN, which was the neuron‐specific marker we used, has a relatively higher abundance in the nucleus than in other cellular compartments. These results clearly indicate that iCAB achieved unbiased biotinylation across various subcellular compartments.

### 2.2 Investigation on Cell‐Type‐Specific Proteome Changes in the Brain of 5xFAD Mice Using iCAB

**Figure 2:**
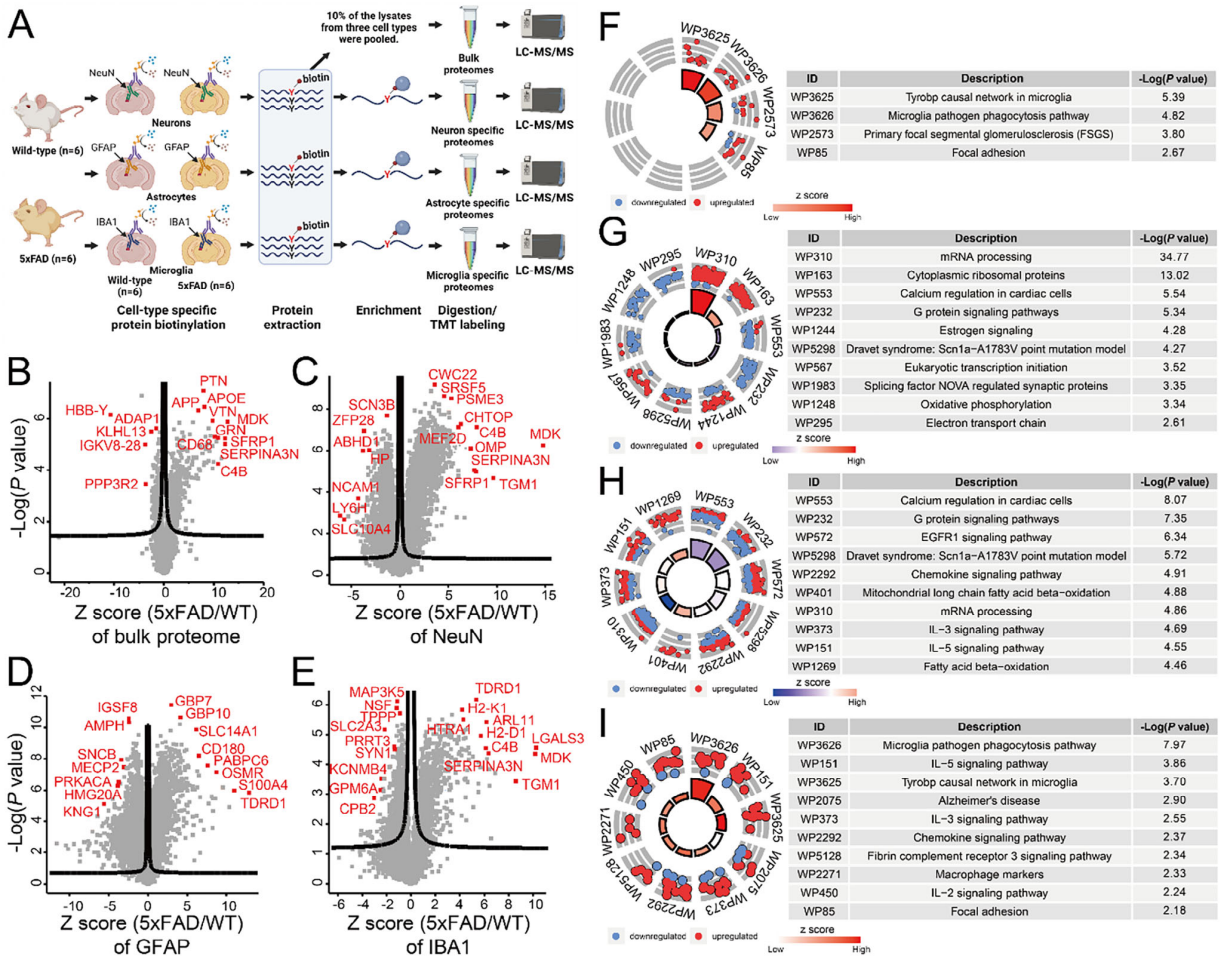
Cell‐type‐specific proteome analysis of 5xFAD mouse brains using iCAB. The iCAB strategy was employed to identify cell‐type‐specific proteomes in the neurons, astrocytes, and microglia of the brains from 6 5xFAD and 6 control mice. To compare the bulk proteome to the enriched ones, 10% of the lysates that were biotinylated for neurons, astrocytes, and microglia were pooled, digested by trypsin, labeled by 12‐plex TMT to multiplex 12 samples, and analyzed by LC‐MS/MS. The rest of the samples were enriched for biotinylated proteins, followed by digesting them by trypsin, labeling them by 12‐plex TMT, and analyzing them by LC‐MS/MS (A). The comparisons between 5xFAD and control mice were conducted for the bulk proteome (B), neuron proteome (C), astrocyte proteome (D), and microglia proteome (E). The proteins outside the curved lines have *q*‐values < 0.05. GSEA of the bulk proteome and cell‐type‐specifically enriched proteomes (F–I). WIKI Pathway analyses using differentially expressed proteins (*q*‐value < 0.01) from the bulk proteomic samples (F) and cell‐type‐specifically enriched samples, including the neurons (G), astrocytes (H), and microglia (I) were conducted. The enriched pathways (*P* value < 0.01) in the WIKI Pathway analysis results were displayed using the GOplot R package. The red and blue circles indicate individual upregulated and downregulated proteins, respectively, in each pathway. The size of the trapezoids in the inner circle of the GO circle corresponds to the –log_10_ (*P* value) of the enriched pathway. The color of the trapezoids corresponds to the Z score. The Z scores are not the standard scores used in statistics, but they are just values to help determine whether a given pathway is likely to be reduced (indicated by a negative value) or enhanced (indicated by a positive value). The Z score can be calculated as follows: Z score = (the number of upregulated proteins – the number of downregulated proteins)/square root (the number of proteins).

Although Aβ overexpression is suspected to be the culprit in the pathogenesis of Alzheimer's disease (AD), how Aβ overexpression changes cell‐type‐specific proteome in the affected brain has not been known yet.^[^ ^15^ ^]^ Thus, applying the iCAB method, we investigated cell‐type‐specific proteome changes in neurons, astrocytes, and microglia from 5xFAD mouse brains, a prominent Aβ overexpression model. We processed cortices and striata from 6 5xFAD and 6 wild‐type (WT) mice using iCAB (**Figure** **2**A). First, we examined the cell‐type‐specific protein biotinylation using chromogen staining, validating cell‐type‐specific protein biotinylation (Figure S8, Supporting Information). To optimize the decrosslinking condition, we tested 4 different decrosslinking buffers and five different reaction times for the PFA‐fixed proteins. The decrosslinking buffer with 4% sodium dodecyl sulfate (SDS) and 300 mm Tris in water after a one‐hour reaction time showed the biggest number of protein identifications, and we used this condition for the follow‐up experiments (Figure S9, Supporting Information). We conducted 4 different batches of 12‐plex TMT experiments to cover samples from bulk proteomics tissue lysate, neurons, astrocytes, and microglia (Tables S8–S11, Supporting Information). From the bulk proteomics tissue lysate, we identified 8164 proteins (7876 proteins with quantity) with 532 upregulated and 398 downregulated proteins in 5xFAD (Figure 2B). From the neurons, we identified 8660 proteins (8444 proteins with quantity) with 2309 upregulated and 2697 downregulated proteins in 5xFAD (Figure 2C). From the astrocytes, we identified 7745 proteins (7613 proteins with quantity) with 2463 upregulated and 2768 downregulated proteins in 5xFAD (Figure 2D). From the microglia, we identified 7976 proteins (7762 proteins with quantity) with 966 upregulated and 736 downregulated proteins in 5xFAD (Figure 2E).

To analyze the dysregulated signaling pathways in the bulk proteomics brain lysate, neurons, astrocytes, and microglia from the 5xFAD mice, we performed gene set enrichment analyses (GSEAs) for the differentially expressed proteins with *q*‐values < 0.01 (Table S12, Supporting Information). The most enriched pathway for the bulk proteomics brain lysate was the Tyrobp causal network in the microglia pathway, followed by the microglia pathogen phagocytosis pathway. The majority of the proteins enriched in the pathways were upregulated in 5xFAD (Figure 2F). The most enriched pathway for the neurons was mRNA processing, followed by cytoplasmic ribosomal proteins. The proteins in these pathways were dominantly upregulated in 5xFAD (Figure 2G). The most enriched pathway for the astrocytes was calcium regulation in the cardiac cell pathway, followed by the G protein signaling pathway. The majority of the proteins in calcium regulation in cardiac cells and G protein signaling pathways were overall downregulated in 5xFAD (Figure 2H). The most enriched pathway for microglia was the microglia pathogen phagocytosis pathway, followed by the IL‐5 signaling pathway. The majority of the proteins in the microglia pathogen phagocytosis pathway and IL‐5 signaling pathway were upregulated (Figure 2I). We also conducted interactome analysis for the proteins in the most enriched pathways. The proteins in the most enriched pathway of the bulk proteomics brain lysate did not form any obvious interactome cluster (Figure S10, Supporting Information). The proteins in the most enriched pathway for neurons formed two different main clusters; mRNA splicing factors formed the biggest cluster, followed by ribosomal proteins (Figure S11, Supporting Information). The proteins in the most enriched pathway for astrocytes formed multiple small clusters, in which PRKACB and GNAO1 were the most connected proteins (Figure S12, Supporting Information). The proteins in the most enriched pathway for microglia formed one small cluster, in which RAC2 and VAV1 were the most connected proteins (Figure S13, Supporting Information). These data demonstrated that the three different cell types showed distinctive proteome profiles and pathways, while the proteome from the bulk proteomics brain lysate only captured a part of the microglia profile. This could be caused by the dilution effect, in which some proteins are differentially expressed only in one cell type and these changes are diluted when brain lysate is analyzed, or the cancel‐out effect, in which the direction of protein expression level in one cell type is opposite of another cell type (Figure S14, Tables S13–S18, Supporting Information). These results suggest that cell‐type‐specific proteome analysis is essential to discover bona fide proteome changes occurring distinctively in each cell type.

### 2.3 Co‐Expression Network Analysis of the Cell‐Type‐Specific Proteome Data from 5xFAD Mouse Brains

So far, we have conducted pathway and interactome analyses using all the differentially expressed proteins. Still, those proteins could be derived from multiple different pathways. Therefore, we needed to dissect them into multiple clusters to sort out highly correlated proteins. This will enable us to have a deeper understanding of the pathway changes in the three different cell types. For this, we carried out a weighted gene co‐expression network analysis (WGCNA) for four proteome data sets from bulk proteomics brain lysate, neurons, astrocytes, and microglia. We then explored the relationship between each module and various traits, such as 5xFAD (Aβ overexpression), sex, AD markers, neuronal subtypes, and astrocyte subtypes.

**Figure 3:**
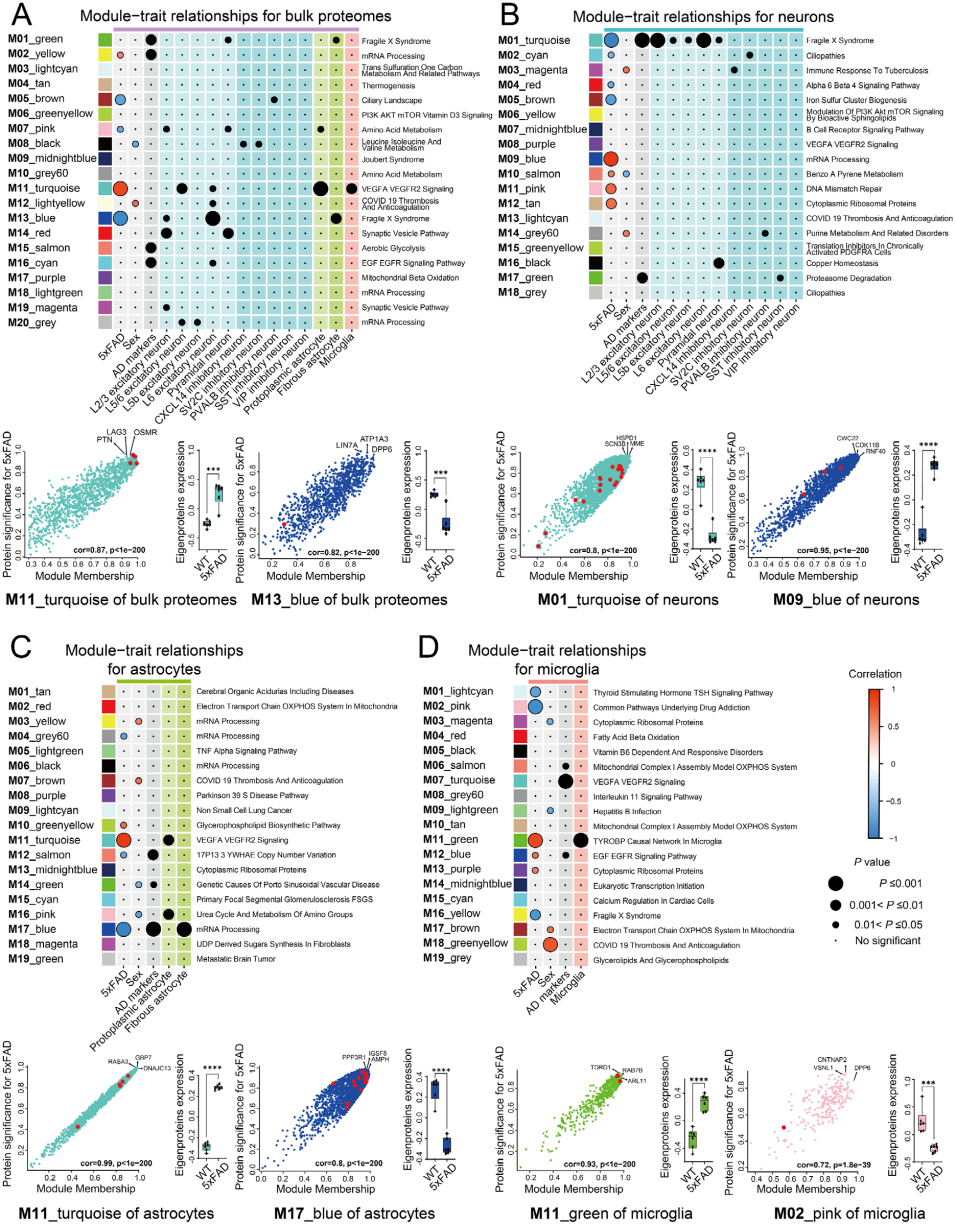
WGCNA of cell type‐specific proteome data. The WGCNA analyses of bulk proteomes (A), neurons (B), astrocytes (C), and microglia (D) data illustrate the connections between modules and traits through bubble charts. The relationships between the protein clusters and 2 traits (5xFAD and sex) were visualized by Pearson correlation. The relationships between the protein clusters and cell types are visualized by the significance of the enrichment. The correlation plots between protein significance (PS) for 5xFAD and module membership (MM) for representative modules are shown for bulk proteomes, neurons, astrocytes, and microglia. Proteins highlighted in red indicate AD markers and names for representative proteins with high PS and MM are shown. An unpaired Student's *t*‐test was used for the statistical analysis (***p < 0.001 and ****p < 0.0001). Whiskers in the boxplots show the full range (min to max) of eigenprotein expression.

WGCNA of the data from bulk proteomics brain lysate identified 20 modules. The M11 module exhibited the most significant positive correlation (upregulation in 5xFAD), whereas the M13 module displayed the most pronounced negative correlation (downregulation in 5xFAD) with 5xFAD traits (**Figure** **3**A top). The M11 module was notably enriched with the markers for L5/6 excitatory neurons, protoplasmic astrocytes, and microglia, as well as with signaling pathways involving VEGFA and VEGFR2. Conversely, the M13 module was enriched with markers of L6 excitatory neurons and fibrous astrocytes, as well as for the Fragile X syndrome pathway (Figure 3A top). Proteins such as, LAG3, OSMR, and PTN were identified as key drivers in the M11 module with increased eigenprotein expression in 5xFAD. Proteins such as LIN7A, ATP1A3, and DPP6 were key drivers in the M13 module with decreased eigenprotein expression in 5xFAD (Figure 3A bottom, Tables S19 and S23 Supporting Information).

WGCNA of the data from neurons identified 18 modules. The M9 module exhibited the most significant positive correlation, whereas the M1 module displayed the most pronounced negative correlation with 5xFAD traits (Figure 3B top). Interestingly, the M1 module was enriched with markers for AD, and L2/3 and L6 excitatory neurons, as well as for fragile X syndrome pathway. On the other hand, the M9 module, which was significantly associated with the mRNA processing pathway, did not show an enrichment for any traits (Figure 3B top). Proteins such as SCN3B, HSPD1, and MME were key drivers in the M1 module with decreased eigenprotein expression in 5xFAD. Proteins such as CWC22, CDK11B, and RNF40 were key drivers in the M9 module with increased eigenprotein expression in 5xFAD (Figure 3B bottom, Tables S20 and S23 Supporting Information).

WGCNA for astrocytes resulted in 19 modules. The M11 module exhibited the most significant positive correlation, whereas the M17 module exhibited the most pronounced negative correlations with 5xFAD traits (Figure 3C top). The M17 module was enriched with markers for AD and fibrous astrocytes, as well as for the mRNA processing pathway. On the other hand, the M11 module was enriched with markers for protoplastic astrocytes, as well as for the VEGFA VEGFR signaling pathway (Figure 3C). Proteins such as RASA3, GBP7, and DNAJC13 were key drivers in the M11 module with increased eigenprotein expression in 5xFAD. Proteins such as PPP3R1, IGSF8, and AMPH were key drivers in the M17 module with decreased eigenprotein expression in 5xFAD (Figure 3C bottom, Tables S21 and S23 Supporting Information).

WGCNA for microglia resulted in 19 modules. The M11 module exhibited the most significant positive correlation, whereas the M2 module exhibited the most prominent negative correlation with 5xFAD traits (Figure 3D top). The M11 module was enriched with markers for microglia, and signaling pathways involving the TYROBP causal network in microglia. Conversely, the M2 module, which is significantly associated with common pathways underlying drug addiction, did not show enrichment for any marker proteins (Figure 3D top). Remarkably, the M18 module associated with COVID‐19 thrombosis and anticoagulation pathway was highly correlated with sex. Proteins such as TDRD1, RAB7B, and ARL11 were key drivers in the M11 module with increased eigenprotein expression in 5xFAD. Proteins such as VSNL1, CNTNAP2, and DPP6 were key drivers in the M2 module with decreased eigenprotein expression in 5xFAD (Figure 3D bottom, Tables S22 and S23 Supporting Information).

These data further demonstrate that the WGCNA results of the bulk proteome exhibit only part of the pathways dysregulated in each cell type of 5xFAD brain, while many key pathway changes are still masked by analyzing mixed cell types, emphasizing the importance of cell‐type‐specific proteome analysis.

### 2.4 Proteome Changes in Subcellular Organelles in Different Cell Types from 5xFAD Mouse Brains

**Figure 4:**
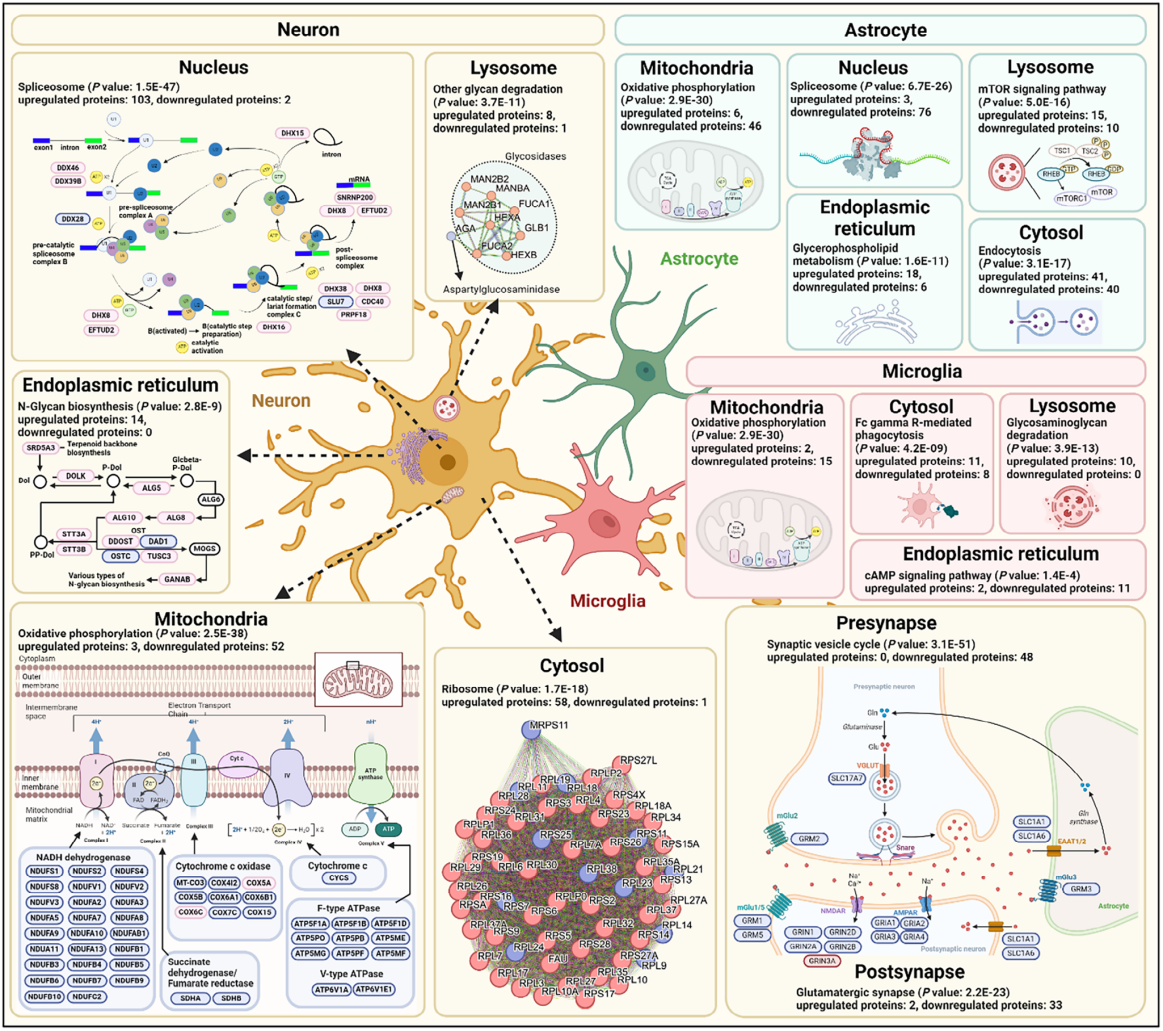
Neuronal and glial proteomic analysis: insights into subcellular dysfunction and pathways in human amyloid‐β overexpression mouse model.

We next investigated the proteome changes in subcellular organelles in three different cell types from 5xFAD mouse brains. We first conducted Gene Ontology Cellular Component (GOCC) to classify proteins based on their subcellular organelle localization (Table S24, Supporting Information). Subsequently, we conducted gene set enrichment analysis for the proteins mapping to respective subcellular organelles (Table S25, Supporting Information). One of the prominent pathways dysregulated in each subcellular organelle of neurons from the 5xFAD mice was as follows: spliceosome for nucleus, other glycan degradation for lysosome, N‐glycan biosynthesis for endoplasmic reticulum (ER), oxidative phosphorylation for mitochondria, ribosome for cytosol, synaptic vesicle cycle for presynapses, and glutamatergic synapse for postsynapse (**Figure** **4**). Most proteins in the oxidative phosphorylation pathway for mitochondria, the synaptic vesicle cycle pathway for presynapse, and the glutamatergic synapse pathway for postsynapse were downregulated in 5xFAD. In contrast, the pathways for other subcellular organelles were upregulated in 5xFAD (Figure 4, Table S26 Supporting Information).

One of the prominent pathways dysregulated in each subcellular organelle of astrocytes from the 5xFAD mice was as follows: spliceosome for nucleus, mTOR signaling pathway for lysosome, glycerophospholipid metabolism for ER, oxidative phosphorylation for mitochondria and endocytosis for cytosol (Figure 4). Most proteins in the glycerophospholipid metabolism pathway for ER were upregulated, whereas most proteins in the oxidative phosphorylation pathway for mitochondria and spliceosome pathway for the nucleus were downregulated (Figure 4, Table S26 Supporting Information).

One of the prominent pathways dysregulated in each subcellular organelle of microglia from the 5xFAD mice was as follows: glycosaminoglycan degradation for lysosome, cAMP signaling pathway for ER, oxidative phosphorylation for mitochondria and Fc gamma R‐mediated phagocytosis for cytosol. No proteins from microglia were enriched for the nucleus (Figure 4). Most proteins in the Fc gamma R‐mediated phagocytosis pathway for cytosol and glycosaminoglycan degradation pathway for lysosome were upregulated. Most proteins in the oxidative phosphorylation pathway for mitochondria and the cAMP signaling pathway for ER were downregulated (Figure 4, Table S26 Supporting Information).

These results demonstrate that each cell type undergoes distinctive pathway changes for most subcellular organelles while sharing some common pathway changes for some subcellular organelles. For example, oxidative phosphorylation for mitochondria was downregulated in three cell types. These data further bolster the importance of cell‐type‐specific proteome analysis.

In the human amyloid‐β overexpressing mouse model, we classified the proteome for the groups of neuron, astrocyte, and microglia into representative subcellular organelles using GO‐CC, such as cytosol, lysosome, mitochondria, nucleus, post/pre‐synapse, and endoplasmic reticulum. The functional and pathway analysis for each subcellular organelle was conducted using the KEGG pathway embedded in Enrichr. The *P* value for the pathway being enriched and the number of up/down‐regulated proteins are noted under the subcellular organelle term.

### 2.5 Cell‐Type‐Specific Expression Profile of the Known AD Marker Proteins

We observed cell‐type‐specifically distinctive expression of the AD marker proteins in Figure 3; AD marker proteins were downregulated in neurons and astrocytes, while mildly upregulated in microglia. To further investigate the cell‐type‐specific expression profile of the AD marker proteins, we grouped them according to the disease progression stages as reported previously and compared their expression changes in 5xFAD in the three cell types.^[^ ^16^ ^]^

**Figure 5:**
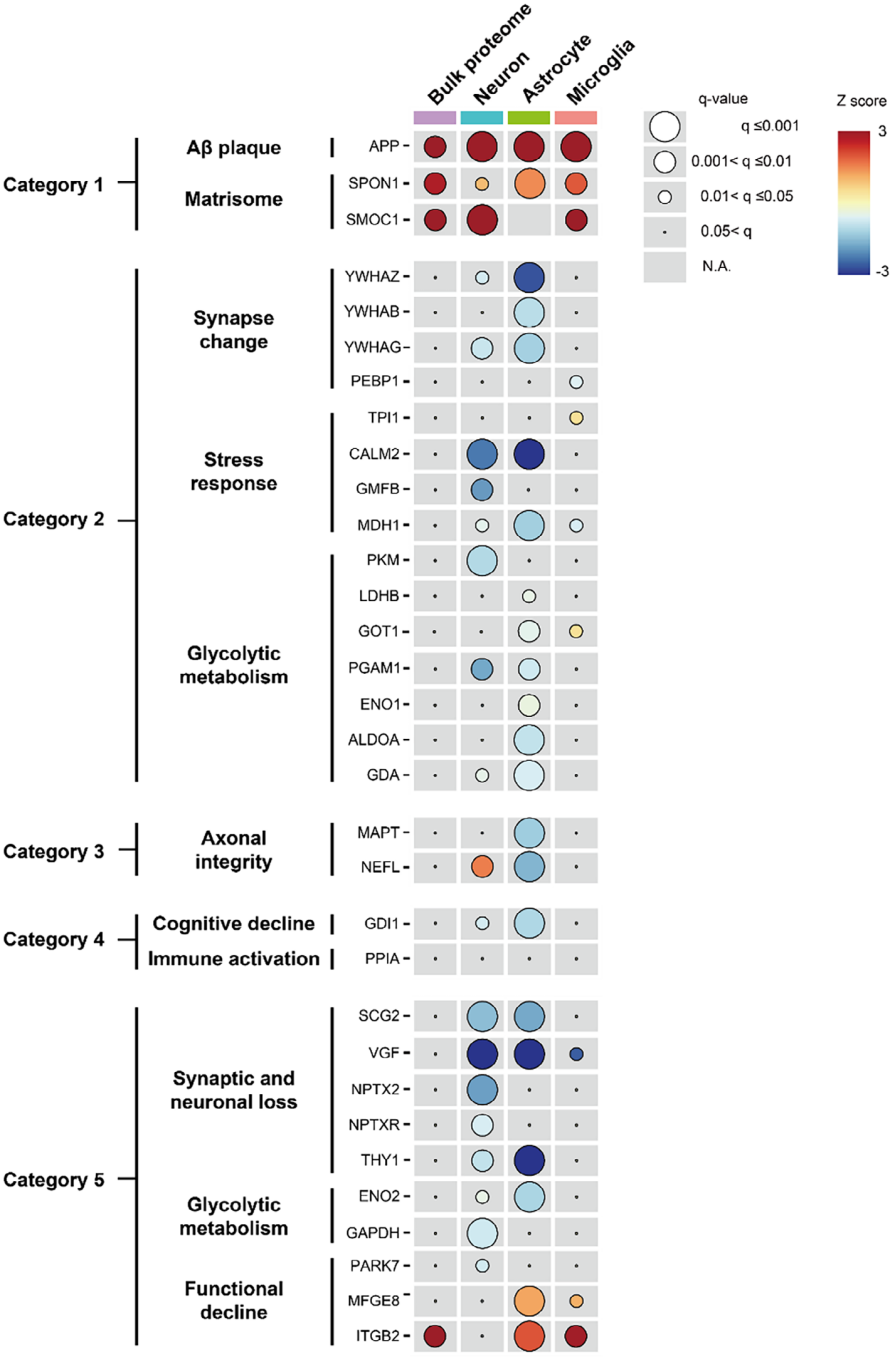
Cell‐type‐specific expression of AD marker proteins in 5xFAD. This bubble plot displays the expression profiles of AD marker proteins across different cell types—bulk proteome, neurons, astrocytes, and microglia—categorized by stages of AD development. The size of each circle corresponds to the *q*‐value, while the color indicates the direction of regulation, represented as a Z‐score. Notably, the SMOC1 protein was not identified in astrocytes, which is why there is no corresponding circle.

Notably, most AD marker proteins were differentially expressed in at least one cell type as assessed by iCAB, whereas most were not in the bulk proteomics data (**Figure** **5**). As expected, the APP expression level was upregulated in all three cell types of 5xFAD. Interestingly, the matrisome proteins SPON1 and SMOC1 were also increased in three cell types, except for SMOC1, which was not identified in astrocytes. Overall, the proteins in Category 2 were downregulated in neurons and astrocytes, while they were upregulated in microglia. In particular, proteins classified for synapse change were more downregulated in astrocytes, the proteins classified for stress response were downregulated in both neurons and astrocytes, and the proteins classified for glycolytic metabolism were more downregulated in astrocytes. Strikingly, MAPT, classified for axonal integrity, was downregulated only in astrocytes, and NEFL, another protein classified for axonal integrity, was increased in neurons and decreased in astrocytes. GDI1, classified for cognitive decline, showed downregulation in neurons and astrocytes. PPIA classified for immune activation did not change in any cell type. All the proteins classified for synaptic and neuronal loss were mostly downregulated in neurons. Three out of 5 proteins classified for synaptic and neuronal loss were also downregulated in astrocytes. ENO2, classified for glycolytic metabolism, was downregulated in neurons and astrocytes, and GAPDH, classified for the same class, was downregulated only in astrocytes. PARK7 protein, classified for functional decline, was downregulated in neurons, and MFGE8 and ITGB2 in the same class were upregulated in astrocytes and microglia. These data demonstrated that the bulk proteomics data do not efficiently manifest changes of actual AD biomarker proteins. On the other hand, most of them changed in at least one of the cell types when assessed with iCAB cell‐type enrichment. This data further emphasizes the importance of cell‐type‐specific proteome analysis.

## 3 Discussion

In this study, we developed a straightforward and user‐friendly approach for cell‐type‐specific proteomics analysis. Using this approach, we investigated the cell‐type‐specific proteome landscape of the 5xFAD human Aβ overexpression mouse model. To achieve cell‐type‐specific proteome analysis, an enzyme that mediates chemical modification of proteins in a target cell type must be localized on the cell type. This has been achieved by expressing mutant‐type tRNA synthase, APEX, HRP, or TurboID^[^ ^17^ ^]^ at target cell types. We accomplished this by using cell‐type‐specific primary antibodies and secondary antibodies to HRP on target cells within a tissue section. When the bulk proteomics result was compared to the cell‐type‐specific analysis one, interestingly, the identified protein number (6213) from the bulk proteomics was smaller than that (8430) of the cell‐type‐specific proteomics. This protein number difference between the bulk proteomics and the enriched cell‐type‐specific proteome data stems from our underestimation of the enrichment efficiency of iCAB. Thus, we injected less amount of peptides into the mass spectrometer for the bulk proteomics, expecting the identified protein number from the iCAB would be quite limited. The data from iCAB comparing the proteomes of three different cell types (Figure 1D–I) revealed differential expressions of almost all proteins in samples enriched specifically for each target cell type. This underscores iCAB's ability to isolate cell‐type‐specific proteomes from the mouse brain effectively. However, we noted marginal enrichment of RBFOX3 (NeuN) when comparing neuronal and astrocyte proteomes. Excessive activation of biotin‐tyramides could possibly cross cell boundaries and biotinylate proteins in neighboring cells. We expect reducing biotin‐tyramide activation levels could control this issue.

We next employed the iCAB method on the 5xFAD mouse model, a widely utilized animal model, to investigate the pathogenesis mechanisms driven by Aβ overexpression in the brain. In this study, we identified ≈8000 proteins from the iCAB‐enriched samples. Since this number is quite close to the number of proteins identified in common brain proteome analysis without cell‐type specific enrichment,^[^ ^18^ ^]^ this large number of protein identifications also bolsters the strength of the iCAB method. Our analysis of the bulk proteomics data revealed only 921 differentially expressed proteins and GSEA of them showed the microglia pathogen phagocytosis pathway was the most dysregulated in 5xFAD, aligning with the previous study.^[^ ^19^ ^]^ On the other hand, remarkably, cell‐type‐specific proteome data revealed 2–5 times more differentially expressed proteins: 5006 in neurons, 5231 in astrocytes, and 1702 in microglia. The differentially expressed proteins in neurons were enriched for mRNA processing. mRNA processing proteins fall into two categories: mRNA splicing factors and ribosomal proteins (Figure S11, Supporting Information). Most of these proteins were related to mRNA splicing. The impaired RNA splicing in Alzheimer's disease (AD) has been reported in multiple studies.^[^ ^20^ ^]^ However, it has been unclear which cell type showed the impaired RNA splicing. Our study indicates that impaired RNA splicing predominantly occurs in neuronal cells.

The differentially expressed proteins in astrocytes were enriched for calcium regulation and G protein signaling pathways. Besides providing trophic and metabolic support, another emerging role of astrocytes is signaling exchange with neurons at tripartite synapses by expressing various neurotransmitter transponders and receptors. G‐protein‐coupled receptor (GPCR) is the most representative receptor. Thereby, astrocytes sense neurotransmitters via GPCRs, leading to increased intracellular calcium. In turn, the activated astrocytes release gliotransmitters such as glutamate, GABA, and ATP/adenosine, activating neurons through neuronal GPCRs.^[^ ^21^ ^]^ Thus, the dysregulated calcium regulation and G protein signaling suggest that the astrocytic function as a synaptic neurotransmission regulator was compromised in the 5xFAD mouse brain. It seems that PRKACB, a mediator of cAMP‐dependent signaling, and GNAO1, a subunit of heterotrimeric G proteins, are key proteins in GPCR‐mediated calcium regulation in astrocytes. The differentially expressed proteins in microglia were enriched for the microglia pathogen phagocytosis pathway. Since microglial activation in the brain of 5xFAD mice is well‐known,^[^ ^22^ ^]^ the activation of microglia was the expected result. Interestingly, our data suggested that Rac2, a small G protein involved in actin and membrane in macrophage, and Vav1, a guanine nucleotide exchange factor, were key proteins in microglial activation.^[^ ^23^ ^]^

WGCNA exhibited a more dissected landscape of cell‐type‐specific proteome change in 5xFAD. The M11 module of the bulk proteome data, which was associated with the VEGFA VEGFR2 signaling pathway, showed a positive correlation with the 5xFAD trait. This pattern was also observed in the M11 module of the astrocyte proteome, suggesting that the upregulated observed in the M11 module of the bulk proteome came from astrocytes. The M13 module of the bulk proteome data, which was associated with the fragile X syndrome pathway, showed a negative correlation with the 5xFAD trait. This was also observed in the M01 module of the neuronal proteome and in the M16 module of the microglia proteome, suggesting the M13 module of the bulk proteome came mostly from the neurons and partially from microglia. Since we have not analyzed other cell types, such as oligodendrocytes, those cell types not analyzed in this study also could have contributed to the bulk proteome. Interestingly, the M09 module of the neuronal proteome and the M17 module of the astrocyte proteome showed an association with the mRNA processing pathway, but the mRNA processing pathway was upregulated in neurons and downregulated in astrocytes of 5xFAD mice. This data suggests that the mRNA processing pathway is activated in neurons, whereas the same pathway is inactivated in astrocytes when these cells are exposed to the stress caused by Aβ overexpression. This could be one of the reasons why the mRNA processing pathway was not enriched in the bulk proteome data. Remarkably, marker proteins for excitatory neurons from the neuron data were downregulated in 5xFAD, while the marker proteins for inhibitory neurons did not change, suggesting that excitatory neurons are preferentially affected by Aβ overexpression. One noticeable observation is L2/3 and L6 excitatory neuronal marker proteins were downregulated with greater statistical significance than other excitatory neuronal marker proteins. Further investigation is required to validate this observation. From the astrocyte proteome, we also observed the marker proteins for fibrous astrocytes, which are mostly found in the white matter, were downregulated, whereas the marker proteins for protoplasmic astrocytes, which are mostly found in the gray matter, were upregulated. This data suggests that fibrous astrocytes are differentially affected by Aβ overexpression than protoplasmic astrocytes.

The subcellular organelle‐dependent proteome change analysis for each cell type gave us deeper insight into the pathway change when each cell type is exposed to Aβ. One of the noticeable pathways not observed in the gene set enrichment analysis conducted with all the differentially expressed proteins was N‐glycan biosynthesis in the endoplasmic reticulum (ER) and the other glycan degradation pathway in the lysosome of the neurons. Most proteins in the pathways were upregulated, suggesting that N‐glycan synthesis in the ER and other glycan degradation in lysosomes were activated. The lysosome in microglia also showed alteration of glycosaminoglycan degradation. There have been multiple reports that glycan is involved in the clearance of Aβ.^[^ ^24^ ^]^ It seems that the glycan synthesis and degradation pathways are activated in neurons and microglia to clear Aβ in 5xFAD mice. We also observed the downregulation of oxidative phosphorylation in the mitochondria of the neurons, astrocytes, and microglia. Neurons and microglia rely on the TCA cycle for energy metabolism, but this TCA cycle shifts to glycolysis as AD advances.^[^ ^25^ ^]^ On the other hand, it is already well known that astrocytes are glycolytic cells, and the stress rendered by Aβ disrupts the glycolytic pathway in astrocytes.^[^ ^26^ ^]^ So, the energy metabolism change cannot be explained by previous studies. Interestingly, the AD marker expression profile in each cell type exhibited that proteins involved in glycolytic metabolism were predominantly downregulated in astrocytes. This data suggests that both glycolytic metabolism and TCA cycle are downregulated in astrocytes of 5xFAD mice. One possible explanation is that the astrocytes in the brain of 5xFAD mice show different energy metabolism changes over the course of aging. Thus, time course study for 5xFAD is required to understand it.

We also observed that proteins classified for synapse change (YWHAZ, YWHAB, and YWHAG) were downregulated in astrocytes, while only YWHAZ and YWHAG were mildly downregulated in neurons. The downregulation of these proteins in neurons can be explained by synapse change mediated by Aβ toxicity. However, the changes for these proteins in astrocytes cannot be explained by this but can be explained by changes in signaling pathways in astrocytes since these three proteins are involved in signal transduction, cell cycle control, and apoptosis.^[^ ^27^ ^]^ Further study is required to understand it.

We also observed that NPTX2 and NPTXR, which are involved in synaptic plasticity,^[^ ^28^ ^]^ are decreased only in neuronal cells. NPTX2 is released from the presynapse of excitatory neurons and binds to the NPTXR expressed on the postsynapse of inhibitory neurons.^[^ ^29^ ^]^ Thereby, NPTX2 activates the inhibitory neurons and, in turn, inactivates the excitatory neurons regulating overexcitation of the excitatory neurons.^[^ ^29^ ^]^ Downregulation of these proteins suggests that excitatory neurons were overstimulated in 5xFAD, leading to their death of excitatory neurons. This data is also in line with the WGCNA results.

Overall, this research underscores the fact that each cell type exhibits distinct proteome changes, many of which have been overlooked in traditional analyses of tissue chunks comprising multiple cell types. Therefore, the conventional bulk analysis approaches may miss critical signaling pathways that are altered during the pathogenesis process. As demonstrated so far, the iCAB method is versatile and can be applied to different cell types in any organ regardless of species, including human tissues, given the availability of a primary antibody that specifically targets the desired cell type. In this context, iCAB can even be applied to formalin‐fixed paraffin‐embedded tissue sections as long as cell‐type‐specific IHC is feasible. In addition, the chromogen‐stained samples showed similar protein abundances to non‐stained samples, indicating that iCAB‐mediated biotinylation can be validated by chromogen staining without compromising proteome quality. More importantly, since iCAB has been developed with a minor modification of IHC by adopting biotin‐tyramide signal amplification, almost all biomedical research laboratories can adapt their IHC to this iCAB without much optimization effort. Once biotinylated, proteins in a target cell type can be easily enriched and identified in general proteomics facilities. This flexibility and user‐friendliness allow its application across diverse disease types, enabling invaluable insights into cell–cell interactions and their function involved in disease pathology.

## 4 Experimental Section

### Preparation of Free‐Floating Mouse Brain Sections

Mouse brains immersed in 4% PFA (Rockland Immunochemical, Gilbertsville, PA, USA) were purchased and prepared mouse brain sections, as reported previously.^[^ ^30^ ^]^ Briefly, mouse brains were washed with 1× cold phosphate‐buffered saline (PBS, Thermo Fisher Scientific, Cleveland, OH, USA) and immersed in 4% cold PFA in 0.1 m phosphate buffer (pH 7.4, Invitrogen, Carlsbad, CA, USA) for 2 days at 4 °C. Subsequently, the brains were incubated with 30% sucrose (Thermo Fisher Scientific) in PBS at 4 °C, allowing them to sink to the bottom of the container completely. Subsequently, the mouse brains were sectioned coronally into 40 µm‐thick slices at −18 to −20 °C using a microtome (Leica Microsystems, Wetzlar, Germany). Floating brain sections (fixed in 4% PFA) were stored in a cryoprotectant solution [ethylene glycol based; 30% ethylene glycol, 30% glycerol, and 10% 0.2 m sodium phosphate buffer pH 7.4, in distilled water (DW)] at −20 °C until use. All reagents related to cryoprotectant solution were purchased from Sigma‐Aldrich (St. Louis, MO, USA).

### Cell‐Type‐Specific Protein Biotinylation Using iCAB

Floating brain sections were selected based on the positions 120 to 317, corresponding to the coronal mouse brain section images provided by the Allen Brain Atlas.^[^ ^31^ ^]^ A total of 1920 coronal brain sections (each 40 µm thick) were pooled and then divided into 16 samples, with each sample containing 120 sections. This division enabled the establishment of four technical replicates (*n *= 4 per group) for each of the four conditions (NeuN, GFAP, IBA1, and negative control). The sections were rinsed twice with 6 mL of PBS in six‐well plates for 5 min each, followed by an additional wash with 6 mL of wash buffer (0.2% Triton X‐100 in PBS) for 10 min at room temperature on a low‐speed rocker. The sections were incubated in 4 mL of blocking buffer (0.2% Triton X‐100 and 5% normal goat serum in PBS) for one hour at room temperature. Triton X‐100, a non‐ionic surfactant included in the blocking buffer, increased cell permeability by disrupting the lipid bilayer and thereby allowed antibodies to access intracellular antigens. Subsequently, primary antibodies were directly added to the floating sections in the blocking buffer at the following dilutions: rabbit anti‐NeuN antibody (Invitrogen, Catalog # PA5‐78499, Polyclonal) at 1:500 for neurons, rabbit anti‐GFAP antibody (Invitrogen, Catalog # PA5‐16291, Polyclonal) at 1:100 for astrocytes, and rabbit anti‐IBA1 antibody (Wako, Catalog # 019–19741, polyclonal) at 1:500 for microglia. The sections treated with the primary antibody were incubated overnight at 4 °C with a low‐speed rocker. Negative control samples were also prepared that were treated in the same way as other samples, except they were not treated with a primary antibody. Each well was washed with 6 mL of wash buffer thrice for 10 min each time and incubated with 5 mL of poly‐HRP‐conjugated secondary antibody solution (SuperBoost Goat anti‐Rabbit Poly HRP, IgG, Invitrogen) for 1 h at room temperature with gentle shaking. Each well was then washed with 6 mL of wash buffer twice for 10 min each time, followed by washing with 6 mL of 0.1 m sodium borate buffer (pH 8.5) thrice for 10 min each time. For cell‐type specific protein biotinylation, the sections were incubated with various concentrations of biotin‐tyramide (Iris Biotech GmbH, Marktredwitz, Germany) in 6 mL of 0.1 m sodium borate (pH 8.5) for 30 min. Each well was washed three times with 6 mL of washing buffer for 10 min each time and incubated in 6 mL of 3% hydrogen peroxide solution for 1 h to deplete the residual HRP from the secondary antibody. Then, the tissue sections were washed with 6 mL of wash buffer thrice for 10 min each time, and the section was incubated with the avidin‐biotin complex (ABC) kit reagent (Vector Laboratories, Burlingame, CA) for 30 min to detect the cell‐type‐specific protein biotinylation of the mouse brain tissue. The ABC kit reagent was prepared by adding 10 µL of avidin and 10 µL of biotin solution to 1 mL of PBS. After washing with 6 mL of wash buffer thrice for 10 min each time, sections were stained using Deep Space Black Chromogen Kit (Biocare Medical, Pacheco, CA, USA). The Deep Space Black solution was prepared by adding 2 µL of deep space black chromogen to 0.5 mL of deep space black buffer per slide. The images of the whole brain sections were acquired using a stereo microscope camera (AmScope, Irvine, CA, USA). The images for the cell‐type‐specifically stained brain cells were acquired on the optical microscope coupled with a 16 MP USB 3.0 Color CMOS C‐Mount Microscope Camera (AmScope).

### Enrichment, on‐Bead Digestion, and Tandem Mass Tagging of Cell‐Type‐Specifically Biotinylated Proteins

The biotinylated tissue sections were lysed with sonication in the lysis buffer (4% SDS, 1% sodium deoxycholate (SDC), and 50 mm of triethylammonium bicarbonate (TEAB) buffer), followed by heating at 99 °C for 1 h. The lysate was then centrifuged at 15 000 × g for 5 min and quantified using BCA analysis, followed by adjusting the protein concentration to 10 mg mL^−1^ by adding the lysis buffer. Subsequently, the lysate was reduced and alkylated using 10 mm tris (2‐carboxyethyl)phosphine hydrochloride (TCEP, Sigma Aldrich) and 40 mm chloroacetamide (CAA, Sigma Aldrich) at room temperature for 1 h. Ten milligrams of proteins per sample were used for the downstream experiment.

To analyze the bulk proteome, 10% of each lysate was aliquoted and subjected to methanol‐chloroform precipitation to remove SDS as follows. The lysate was mixed with 4 volumes of 100% methanol and briefly vortexed. Subsequently, 1 volume of chloroform was added, followed by vortexing. After adding 3 volumes of DW, phase separation was induced by centrifugation at 12 000 × g for 10 min. The upper and lower layers were carefully removed, and the precipitated protein layer was washed with 500 µL of methanol. The washed protein layer was then digested with trypsin (sequencing grade modified trypsin; Promega) in a mixture of 100 mm ammonium bicarbonate (pH 8) and 2 m urea at the ratio of 50:1 for protein to trypsin. The digestion was carried out at 37 °C overnight. The resulting peptides were desalted with C18 StageTips (3M Empore, St. Paul, MN, USA).

To enrich the biotinylated proteins, the remaining 90% of the lysate was reduced and alkylated then mixed with 1 volume of 50 mm TEAB to dilute 4% SDS to 2% SDS. The diluted lysate was incubated with ≈340 µL of streptavidin‐sepharose beads (GE Healthcare, #17‐5113‐01) at room temperature for 2 h with gentle rotation. Subsequently, the supernatant was removed from the beads, and the beads were washed five times with washing buffer 1 (1% Triton X‐100 and 0.2% SDS in DW). The beads were subsequently washed twice with washing buffer 2 (50 mm ammonium bicarbonate in DW). The biotinylated proteins bound to the beads were digested with sequencing‐grade trypsin (Promega, Madison, WI, USA) at the ratio of 50:1 for protein to trypsin in 100 mm ammonium bicarbonate (pH 8) and 2 m urea at 37 °C overnight. The digested peptides were collected and desalted with strong cation exchange (SCX) Stage‐Tips (3M Empore). Both the digested peptides from bulk proteomic and enriched samples were labeled with 16‐plex TMT according to the manufacturer's instructions (Thermo Fisher Scientific). When the 16 samples were labeled by TMT, the order of the samples was randomized to avoid any potential experimental bias. Briefly, the labeling reaction was carried out at room temperature for 1 h, followed by quenching with 1/10 volume of 1 m Tris–HCl (pH 8.0). The obtained TMT‐labeled peptides were pooled, vacuum dried using a SpeedVac (Thermo Fisher Scientific) and then stored at −80 °C until use.

To identify biotinylated peptides derived from the biotinylated proteins, the biotinylated peptides that remained bound to the streptavidin beads after on‐bead digestion were eluted as follows. After on‐bead digestion, the streptavidin beads were washed four times with PBS. Then, the elution of the biotinylated peptides was conducted at 70 °C using an 80% ACN solution containing 0.2% formic acid (FA) and 0.1% TFA. After desalting, the enriched biotinylated peptides were reconstituted in 0.5% FA and analyzed using mass spectrometry.

### Pre‐Fractionation of Peptides

The cell‐type‐specific TMT‐labeled peptides were reconstituted in 10 mm TEAB and underwent pre‐fractionation using bRPLC, fractionating them in 96 fractions. Subsequently, these 96 fractions were concatenated into 24 fractions. For the bRPLC fractionation, an Agilent 1260 offline LC system (Agilent Technologies) was employed, which consisted of a binary pump, a UV detector, an autosampler, and an automatic fraction collector. Briefly, the dried peptide samples were reconstituted in solvent A (10 mm TEAB in water, pH 8.5) and loaded onto an Agilent 300 Extend‐C18 column (5 µm, 4.6 mm × 25 cm; Agilent Technologies). The peptides were then resolved using an increasing gradient of solvent B [10 mm TEAB in 90% acetonitrile (ACN), pH 8.5]) at a flow rate of 0.3 mL min^−1^, with a total run time of 150 min. Finally, the 24 concatenated samples were vacuum‐dried using a SpeedVac and stored at −80 °C until further use.

### Mass Spectrometry Analysis

Liquid chromatography with tandem mass spectrometry (LC‐MS/MS) analysis was conducted as described previously with minor modifications.^[^ ^32^ ^]^ The Orbitrap Fusion Lumos Tribrid mass spectrometer (Thermo Fisher Scientific) coupled with an Ultimate 3000 RSLCnano nanoflow liquid chromatography system (Thermo Fisher Scientific) was used to analyze the prepared peptides. The peptides from each fraction were reconstituted in 0.5% FA and loaded onto a trap column (Acclaim PepMap 100, LC C18, 5 µm, 100 µm × 2 cm, nanoViper) at a flow rate of 8 µL min^−1^. The peptides were resolved at a flow rate of 0.3 µL min^−1^ using an increasing gradient of solvent B (0.1% FA in 95% ACN) on an analytical column (Easy‐Spray PepMap RSLC C18, 2 µm, 75 µm × 50 cm) equipped with an EASY‐Spray ion source that was operated at a voltage of 2.4 kV. The total run time was 120 min. Mass spectrometry (MS) analysis was performed in data‐dependent acquisition mode with a full scan in the mass‐to‐charge ratio (*m/z*) range of 300 to 1800 in the “Top Speed” mode with 3 s per cycle. The precursor mass (MS1) and fragment mass (MS2) scans were acquired for the precursor and the peptide fragmentation ions, respectively. MS1 scans were measured at a resolution of 120 000 at an *m/z* 200, while MS2 scans were acquired by fragmenting precursor ions using the higher‐energy collisional dissociation (HCD) method, which was set to 35% and detected at a mass resolution of 50 000 at an *m/z* 200. The automatic gain control targets were set to one million ions for MS1 and 0.05 million ions for MS2. The maximum ion injection time was set to 50 ms for MS1 and 100 ms for MS2. The precursor isolation window was set to 1.6 *m/z* with 0.4 *m/z* of offset. Dynamic exclusion was set to 30 s, and singly charged ions were rejected. Internal calibration was carried out using the lock mass option (*m/z* 445.12 002) from ambient air.

### Database Searches for Cell‐Type Specific Proteomes

Database searches were conducted as described previously with minor modifications.^[^ ^32^ ^]^ The MS/MS data obtained from LC‐MS/MS analyses were used to search against the UniProt mouse protein database (UP000000589, included both Swiss‐Prot and TrEMBL and released in January 2019 with 55 435 entries), which contained protein entries with common contaminants (115 entries), using MSFragger 3.4 search algorithms through Thermo Proteome Discoverer software suite (version 2.4.1.15, Thermo Scientific).^[^ ^33^ ^]^ The top ten peaks in each window of *m/z* 100 were selected for database search during MS2 preprocessing. The following parameters were used for the database search. Trypsin was set as the protease, allowing a maximum of two missed cleavages. Carbamidomethylation of cysteine (+57.02 146 Da), TMTpro tags (+304.20 715 Da) on lysine and peptide N termini were set as fixed modifications, while oxidation (+15.99 492 Da) of methionine and biotinylation (+361.14 600 Da) of tyrosine were set as variable modifications. The minimum peptide length was set to six amino acids. The MS1 and MS2 tolerances were set to 10 and 20 ppm, respectively. Peptides and proteins were filtered at a 1% false discovery rate (FDR) using the percolator node and protein FDR validator node, respectively. The following parameters were used for the protein quantification. The most confident centroid option was used for the integration mode, while the reporter ion tolerance was set to 20 ppm. The MS order was set to MS2, and the activation type was set to HCD. Both unique and razor peptides were used for peptide quantification, while protein groups were considered for peptide uniqueness. The co‐isolation threshold was set to 50%. Reporter ion abundance was computed based on signal‐to‐noise ratios, and the missing intensity values were replaced with the minimum value. The average reporter signal‐to‐noise threshold was set to 10. The quantification value corrections for isobaric tags and data normalization were disabled. Protein grouping was performed with a strict parsimony principle to generate the final protein groups. All proteins sharing the same set or subset of identified peptides were grouped, while protein groups with no unique peptides were filtered out. Proteome Discoverer iterated through all spectra and selected PSMs with the highest number of unambiguous and unique peptides, and then final protein groups were generated. The Proteome Discoverer summed all the reporter ion abundances of PSMs for the corresponding proteins.^[^ ^34^ ^]^

### Statistical Analysis

The statistical analysis of the mass spectrometry data was performed with the Perseus software (version 1.6.0.7).^[^ ^35^ ^]^ For normalization, the reporter ion intensity values were divided by the median values of each protein, and the relative abundance values for each sample were subtracted by the median values of each sample after the log_2_ transformation. Subsequently, a z‐score transformation was applied to the data. The *P* values between the comparison groups were calculated using the Student's two‐sample *t*‐test. Proteins with *q*‐values < 0.05 were considered differentially expressed. Additional details about the number of samples (*N*) and replicates have been included in the figure legends.

### Bioinformatics Analysis

The *q‐*values for the volcano plot were calculated by SAM and a permutation‐based FDR estimation with 0.1 of the S0 value.^[^ ^36^ ^]^ The Azimuth database embedded in Enrichr was used to discover the closest cell type for the input list of genes.^[^ ^37^ ^]^ Azimuth was an annotated reference dataset constructed based on single‐cell RNA‐seq data.^[^ ^38^ ^]^ The development of Azimuth was led by the New York Genome Center Mapping Component as part of the NIH Human Biomolecular Atlas Project (HuBMAP).^[^ ^39^ ^]^ The top 300 upregulated proteins with *q*‐values < 0.05 were used from a specific cell type for the Azimuth cell‐type analysis. PCA and heatmap analyses were conducted using the MetaboAnalyst tool.^[^ ^40^ ^]^ Gene Ontology Cellular Component (GO‐CC) analysis embedded in DAVID bioinformatics resources (version 6.8) was used to examine the subcellular distribution pattern of the proteins identified from each cell type.^[^ ^41^ ^]^ For the GO‐CC analysis, all the proteins identified by three or more peptides were used. Jvenn was utilized to generate Venn diagrams comparing different groups to evaluate non‐specific binding proteins.^[^ ^42^ ^]^

### Application of the iCAB to 5xFAD Transgenic Mice

This research complied with all relevant ethical regulations. All animal experiments were according to the guidelines of the Laboratory Animal Manual of the National Institute of Health Guide to the Care and Use of Animals and were approved by the Johns Hopkins Medical Institute Animal Care and Use Committee (Protocol Number: MO23M244).^[^ ^43^ ^]^

iCAB was employed on the cell‐type‐specific analysis of 12‐month‐old 5xFAD and WT mice. The brain sections used in this study corresponded to positions 120 to 317 of the coronal mouse brain sections in the Allen Brain Atlas.^[^ ^31^ ^]^ The sample size was determined using the pwr package in *R*, as described previously, with minor modifications.^[^ ^32^ ^]^ When aiming to detect proteins with 1.5‐fold differences between the groups, the minimum required sample size was calculated to be 5.928, with a significance level of 0.0005, power of 0.8, sigma (*σ*) of 0.162 (derived from the in‐house TMT proteomics experiments), and delta (*Δ*) of 0.585 (corresponding to log_2_(1.5)). The significance level of 0.0005 was determined based on our in‐house data. When several thousand proteins were identified, most of those with *P* values < 0.0005 exhibited *q*‐values < 0.05. Thus, it was decided to use 6 mice per group. The 12 mouse brains were subjected to iCAB with minor modifications as follows. Before starting the iCAB analysis of the mouse brain samples, the best conditions for the decrosslinking of PFA‐modified proteins were determined. For this, four different decrosslinking buffers were evaluated for the PFA‐modified proteins: 1) 4% SDS, 1% SDC, and 50 mm of TEAB; 2) 4% SDS, 300 mm Tris, 1% SDC, and 50 mm of TEAB; 3) 4% SDS, 300 mm Tris, 1% SDC, 25 mm TEAB, and 50% ACN; 4) 4% SDS, 300 mm Tris, 1% SDC, 25 mm TEAB, and 50% tetrafluoroethylene (TFE). Decrosslinking was conducted using the aforementioned four different buffers, followed by heating at 99 °C for various reaction times of 1, 3, 6, 12, and 24 h.^[^ ^44^ ^]^ Four batches of 12‐plex TMT experiments were conducted to cover neurons, astrocytes, microglia, and bulk proteomic samples. To enrich cell‐type‐specifically biotinylated proteins, 4.9 mg of proteins were used for the starting material. The rest of the enrichment, TMT labeling, mass spectrometry analysis, and data analysis were conducted as described above. The bulk proteomic samples were prepared by pooling 200 µg of peptides prepared for neurons, astrocytes, and microglia, followed by TMT labeling of them. Subsequently, the TMT‐labeled peptides were pooled and prefractionated into 24 fractions. The rest of the procedures were conducted in the same way as described above for the comparison between cell‐type‐specifically enriched proteomes. For bioinformatics analysis, the WIKI pathway integrated into DAVID was used, and the results were visualized using the GO plot package in R.^[^ ^45^ ^]^ Furthermore, the interactome was analyzed using STRING‐PPI database version 12.^[^ ^46^ ^]^ WGCNA was performed using the R software package.^[^ ^47^ ^]^ The minimum soft threshold value that reaches 0.9 was used for Scale Free Topology Module Fit using a signed network to generate dendrograms (Figure S15, Supporting Information). WIKI pathway analyses were conducted with proteins in each module to figure out enriched pathways for them. A database of deeply integrated human single‐cell omics data was employed to construct a database for cell‐type‐specific marker genes (Subtype DB).^[^ ^16^ ^]^ For this, the list of genes annotated was compared with each brain cell type and removed genes that were found in more than one cell type. The *P* values for the correlations between the subtype DB genes or AD marker proteins and module genes were calculated using Fisher's exact test. For the GSEA of the subcellular organelle proteome from three cell types, differentially expressed proteins were classified based on GO‐CC categories, and proteins classified for each subcellular organelle were subjected to EnrichR.

## Conflict of Interest

The authors declare no conflict of interest.

## Author Contributions

T.R., S.Y.K., T.T., and C.H.N. designed the experiments. T.R., S.Y.K., T.T., J.S., and Y.J. performed the experiments. J.S. and T.I.K. provided 5xFAD mice. T.R. and S.Y.K. analyzed the data. T.R., S.Y.K., T.T., Y.J., J.S., T.I.K., and C.H.N. wrote the manuscript. C.H.N. supervised the research.

## Supporting information



Supporting Information

Supplemental Tables

## Data Availability

The data that support the findings of this study are openly available in ProteomeXchange at https://www.proteomexchange.org, reference number PXD046362.
