# Supplementary material for: In‐Depth Cell‐Type‐Specific Proteome Landscape of the Brain from Human Amyloid‐β Overexpression Mouse Model
Source: Adv Sci (Weinh). 2025 May 8;12(20):2409318. doi: 10.1002/advs.202409318 (PMC12120748; doi:10.1002/advs.202409318)
Supplement: Supplementary file 1 — Supporting Information [file ADVS-12-2409318-s001.docx]

Supporting Information

**In-Depth Cell-Type-Specific Proteome Landscape of the Brain from Human Amyloid-β Overexpression Mouse Model**

Taekyung Ryu^1,2,†^, Seok-Young Kim^1,2,†^, Thujitha Thuraisamy^1,2^, Jisu Shin^1,2^, Yura Jang^1,2^, Tae-In Kam^1,2,3^, Chan Hyun Na^1,2,^*

^1^Department of Neurology, Johns Hopkins University School of Medicine, Baltimore, Maryland, 21205, USA

^2^Neuroregeneration and Stem Cell Programs, Institute for Cell Engineering, Johns Hopkins University School of Medicine, Baltimore, Maryland, 21205, USA

^3^Department of Brain and Cognitive Sciences, Korea Advanced Institute of Science and Technology, Daejeon, 34141, South Korea

* For correspondence: Chan Hyun Na, [chanhyun@jhmi.edu](mailto:chanhyun@jhmi.edu)

^†^ These authors contributed equally to this work.


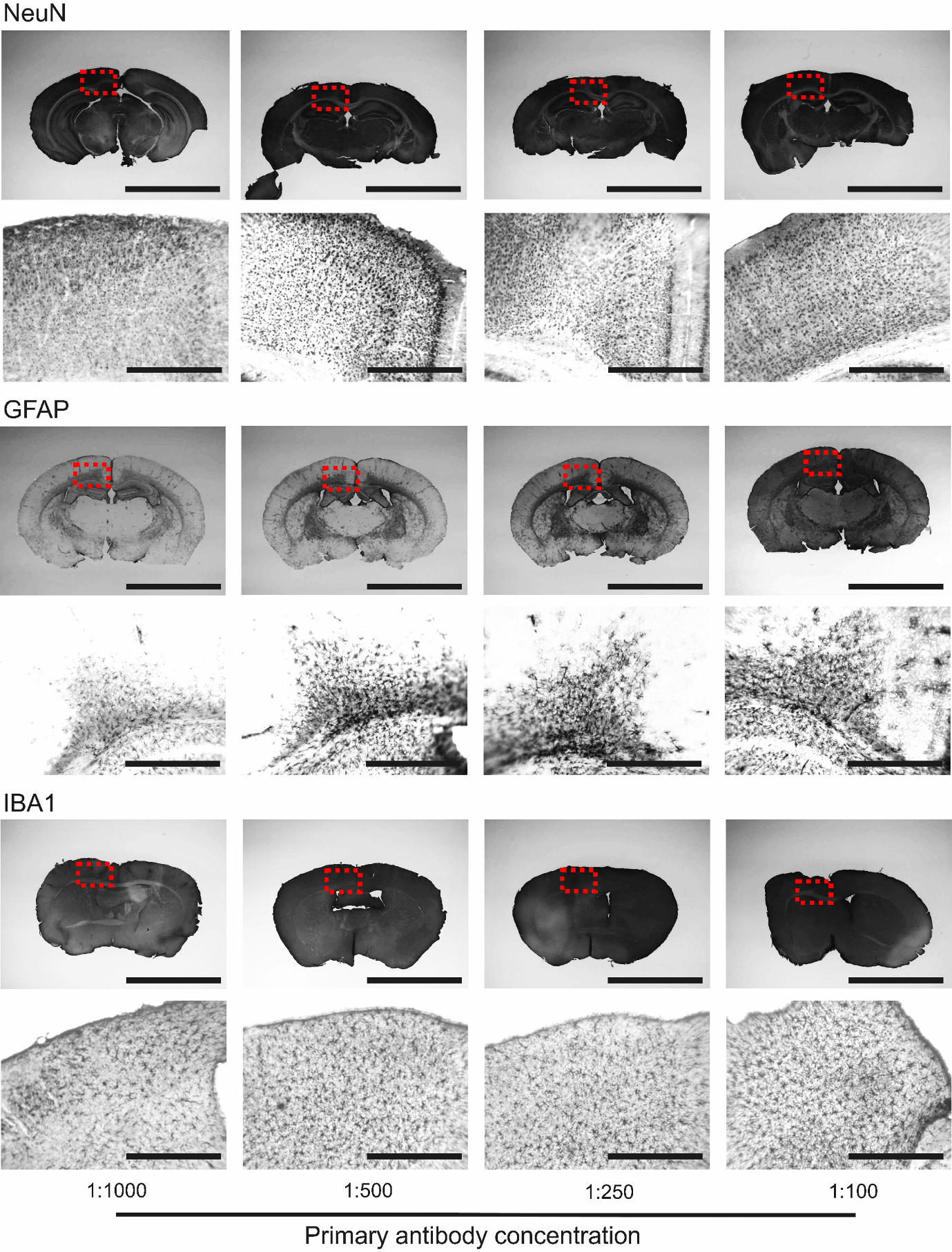


**Figure S1. Optimization for the primary antibody concentrations of the iCAB method.**

Different concentrations of anti-NeuN antibody (NeuN), anti-GFAP antibody (GFAP), and anti-IBA1 antibody (IBA1) were tested for iCAB. The images on the top are of whole brain sections, and the images on the bottom are zoom-ins of the red dotted squares in the images on the top. The lengths of the scale bars for the whole sections (top) and the F-P cortices (bottom) are 5 mm and 500 µm, respectively.

**
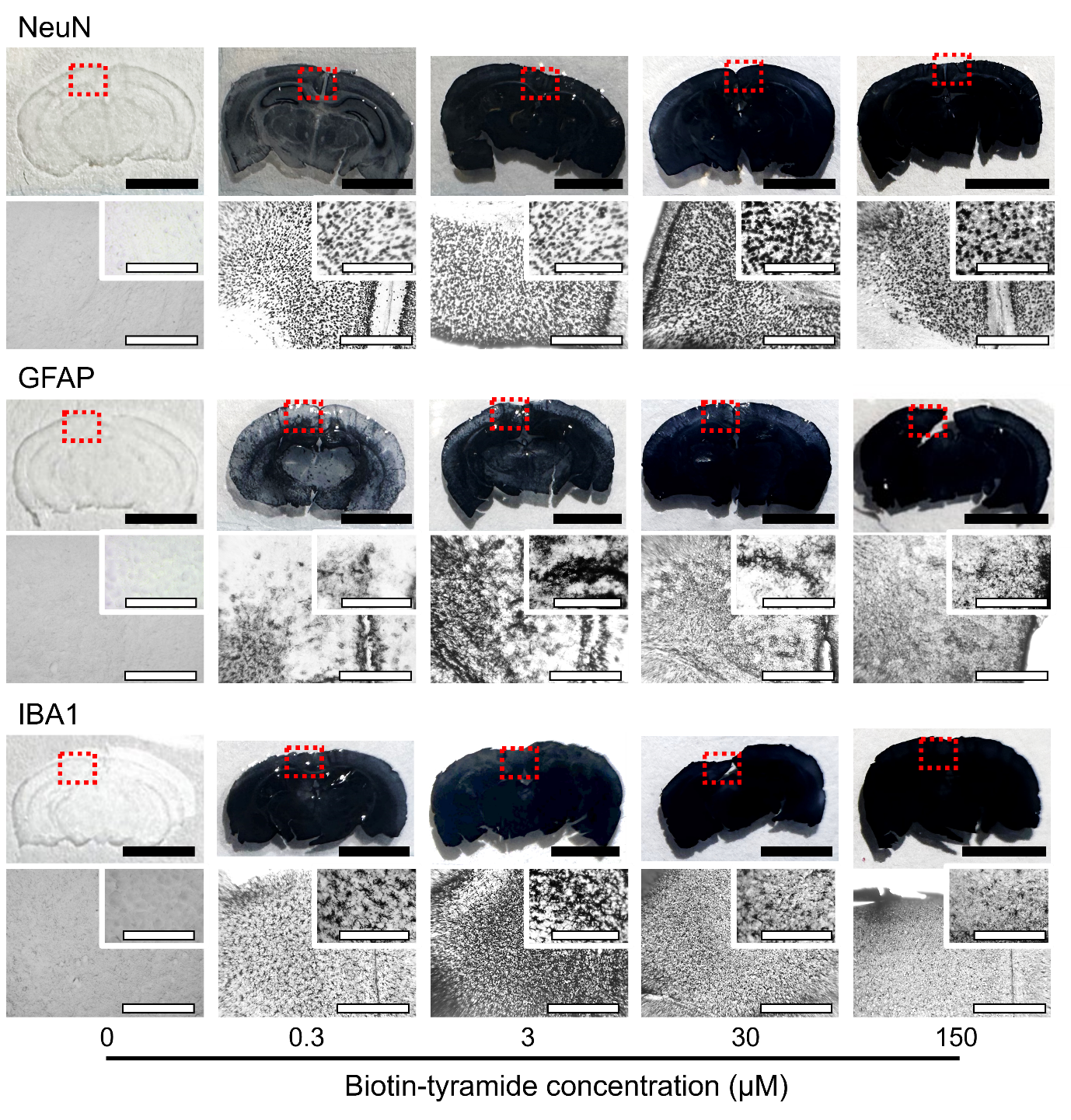
**

**Figure S2. Optimization for the biotin-tyramide concentrations of the iCAB method.**

Five different concentrations of biotin-tyramide for anti-NeuN antibody (NeuN), anti-GFAP antibody (GFAP), and anti-IBA1 antibody (IBA1) were evaluated to find the optimal biotin-tyramide concentrations for them. The images on the top are of whole brain sections, and the images on the bottom are of zoom-ins of the red dotted squares in the images on the top. The lengths of the scale bars for the whole sections (top), P-F cortices (bottom), and the insets of P-F cortices (the top-right corner of the images on the bottom) are 5 mm, 500 µm, and 100 µm, respectively.


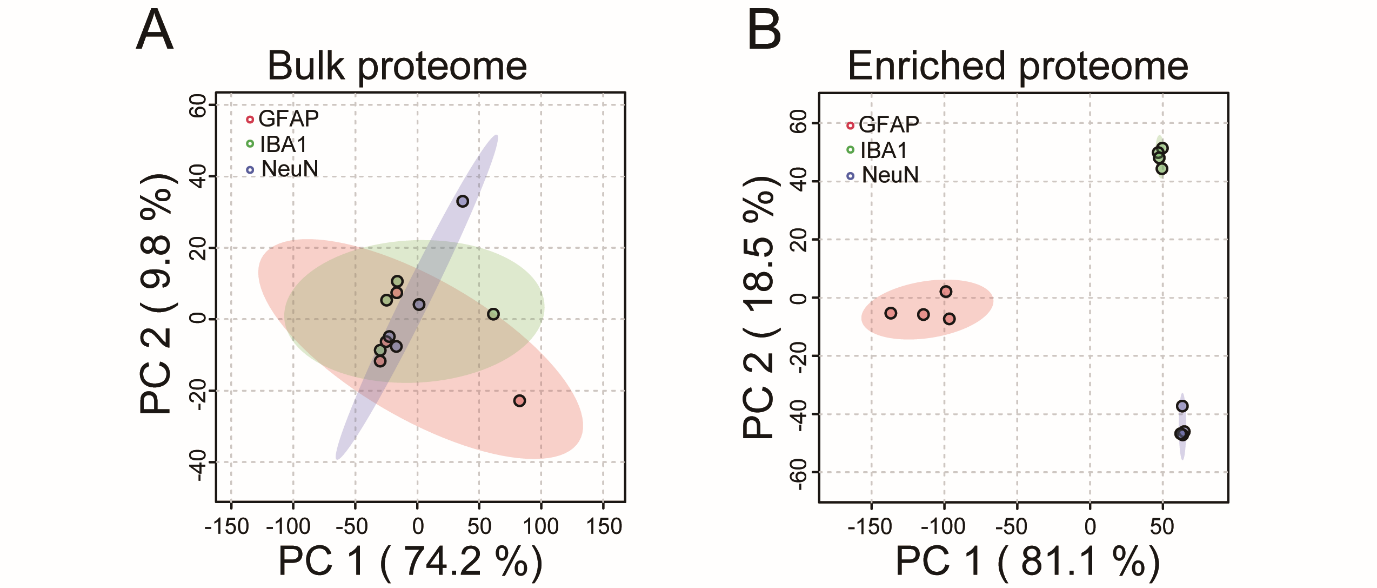


**Figure S3. Principal component analysis (PCA) of the cell-type specific proteome data by iCAB.**

The PCA for the bulk (A) and cell-type specifically enriched (B) proteome data were conducted. GFAP, IBA1 and NeuN (n=4, technical replicates for each group) represent the proteome data for astrocytes (anti-GFAP antibody), microglia (anti-IBA1 antibody), and neurons (anti-NeuN antibody).

**
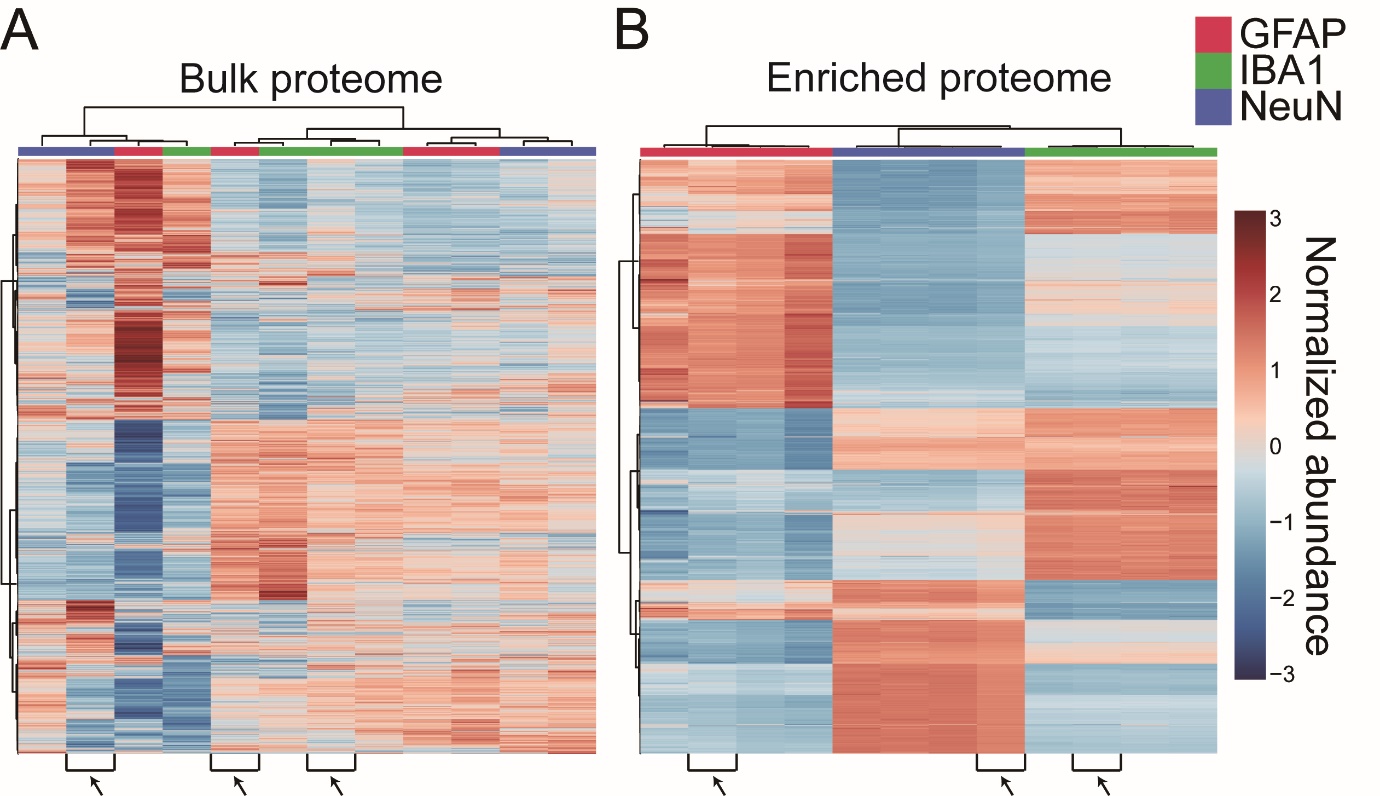
**

**Figure S4. Hierarchical clustering analysis.**

Hierarchical clustering analyses for bulk proteomic (A) and cell-type specifically enriched (B) samples were conducted. GFAP, IBA1, and NeuN (n=4, technical replicates for each group) represent the proteome data for astrocytes (anti-GFAP antibody), microglia (anti-IBA1 antibody), and neurons (anti-NeuN antibody). The columns indicated by the arrow at the bottom of the heatmap refer to samples stained with chromogen.


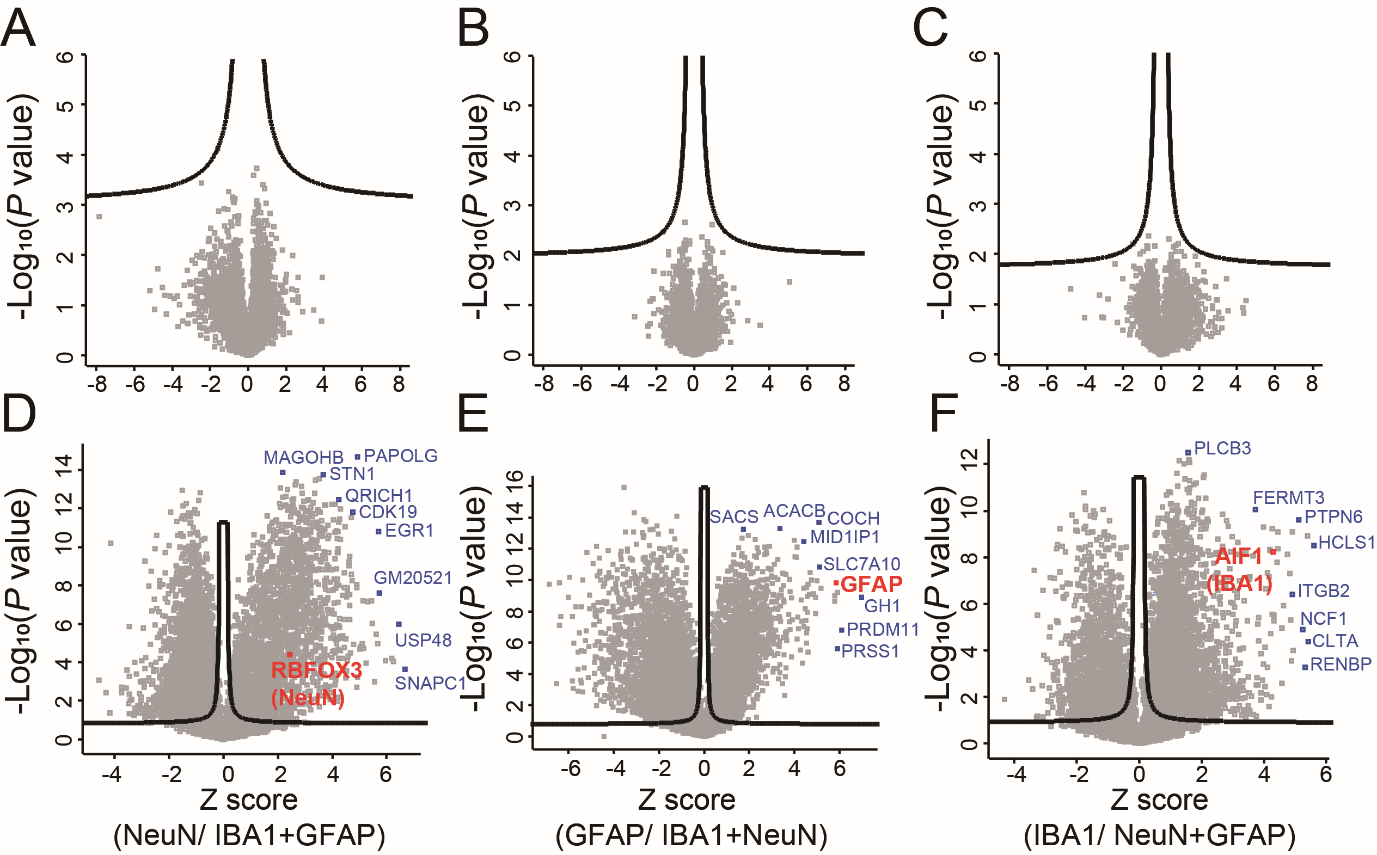


**Figure S5. Volcano plot analysis to estimate the cell-type-specific enrichment of one cell type compared to two other cell types.**

The three volcano plots on the top (A-C) are of bulk proteome data from the samples (n=4, technical replicates for each group), which were processed for iCAB but not enriched. The three volcano plots on the bottom (D-F) are of cell-type-specific proteome data (n=4, technical replicates for each group), which were processed for iCAB and enriched for biotinylated proteins. The proteome data of anti-NeuN-antibody (NeuN; A and D), anti-GFAP antibody (GFAP; B and E), and anti-IBA1 antibody (IBA1; C and F) were compared to that of the other two cell types. The proteins outside the curved lines have q-values < 0.05. An unpaired Student’s t-test was used for the statistical analysis.


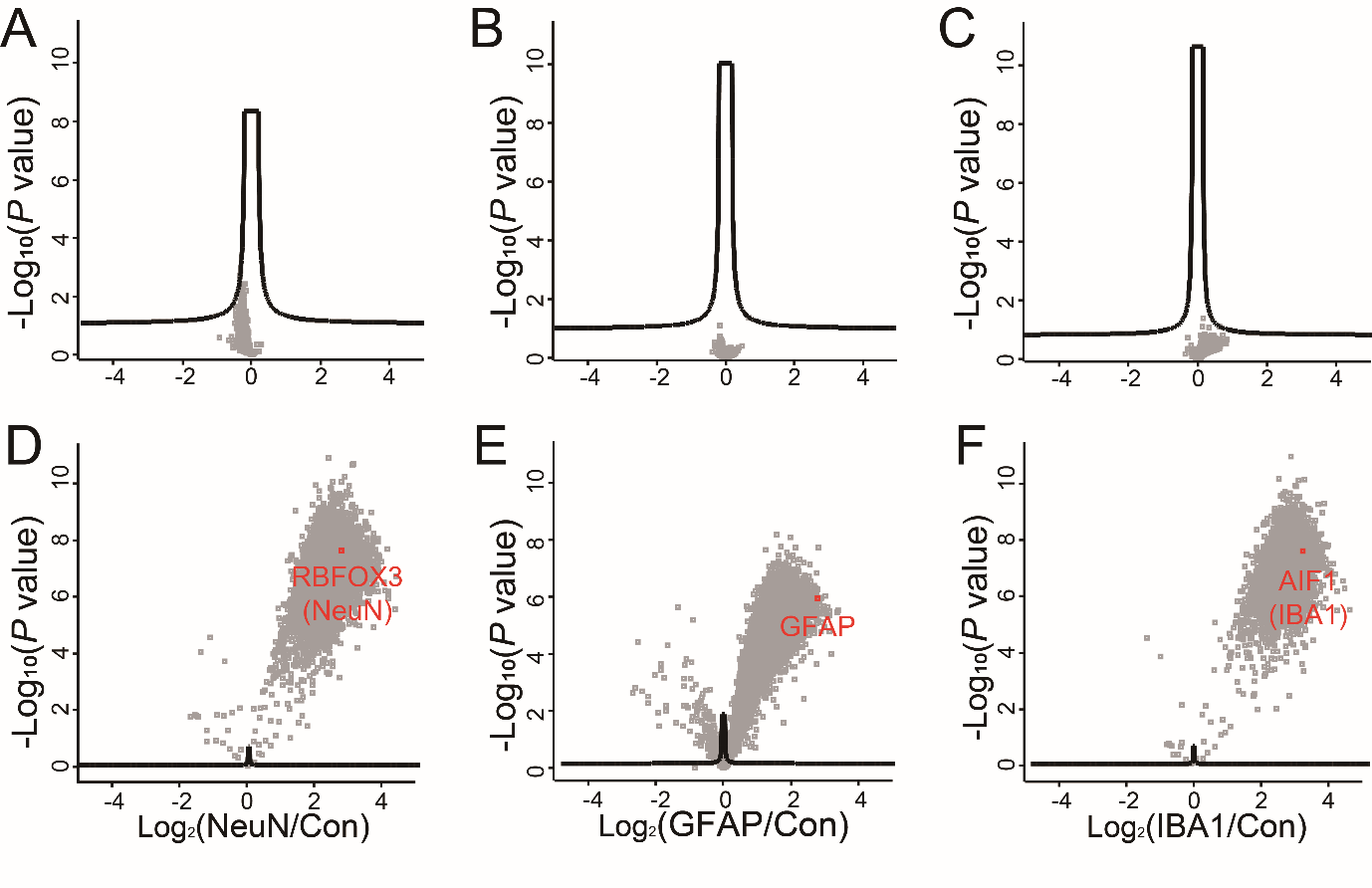


**Figure S6. Volcano plots comparing bulk proteomes or cell-type specifically enriched proteomes with a negative control sample not treated with the primary antibodies.**

The three volcano plots on the top (A-C) represent the bulk proteome of the cell-type-specifically biotinylated samples without or without primary (n=4, technical replicates for each group). The three volcano plots on the bottom (D-F) represent cell-type specific proteomes prepared by the iCAB method with or without the primary antibodies. The cell-type specific proteome data prepared by iCAB using anti-NeuN antibody (NeuN; A and D), anti-GFAP antibody (GFAP; B and E), and anti-IBA1 antibody (IBA1; C and F) were compared to that of the negative control, which was not treated with a primary antibody (n=4, technical replicates for each group). Proteins outside the curved lines have q-values < 0.05. These data were not normalized across the samples, as the protein amounts from the control group were expected to be significantly lower than those from the other groups. An unpaired Student’s t-test was used for the statistical analysis.


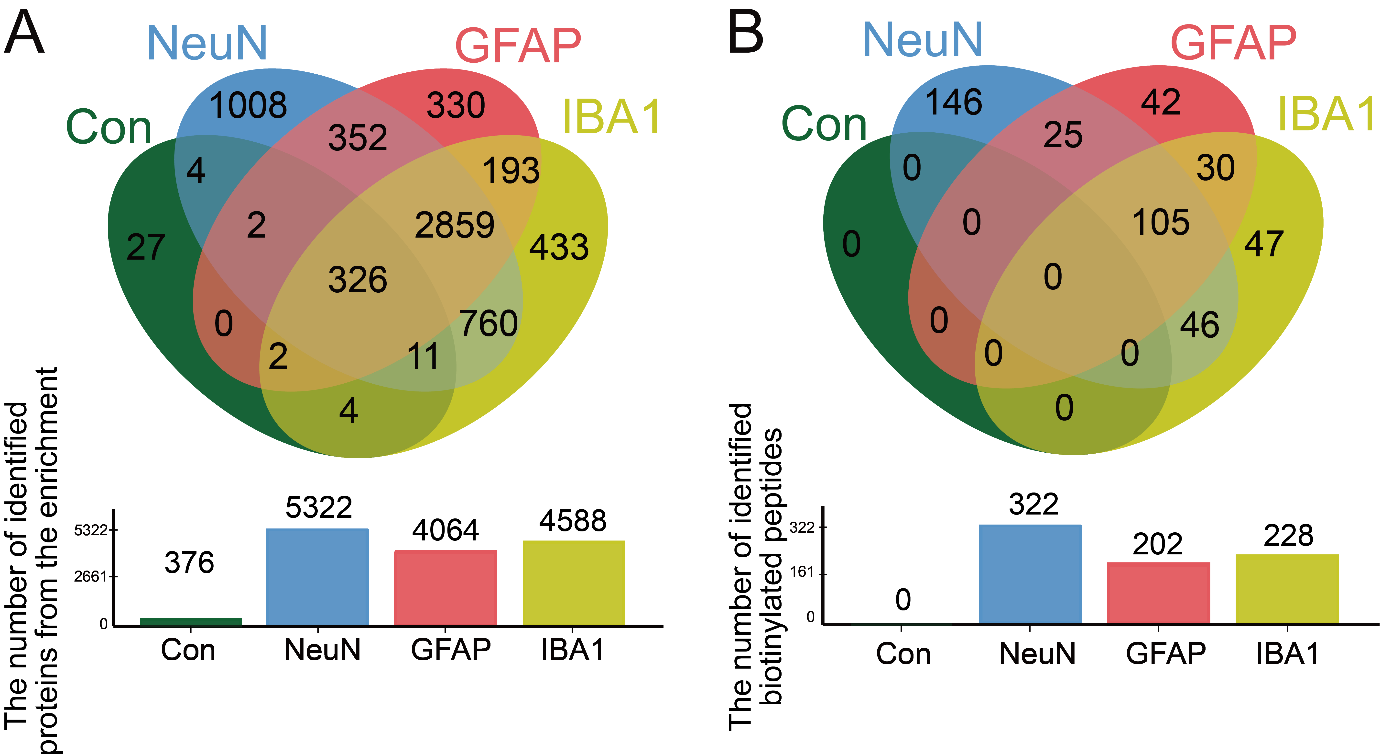


**Figure S7. The numbers of proteins and biotinylated peptides identified from each cell type and negative control.**

The Venn diagrams illustrate the overlap of identified proteins (A) and peptides (B) among the negative control (Con, green), NeuN (neurons, blue), GFAP (astrocytes, red), and IBA1 (microglia, yellow) (n=4, technical replicates for each group). The bar graphs illustrate the number of identified proteins (A) and biotinylated peptides (B) from the negative control (Con, green), NeuN (neurons, blue), GFAP (astrocytes, red), and IBA1 (microglia, yellow) (n=4, technical replicates for each group). The label-free cell-type-specific protein data (A) were from the one used for Figure 1K. The negative control represents the group in which no antibodies were applied to the tissue.


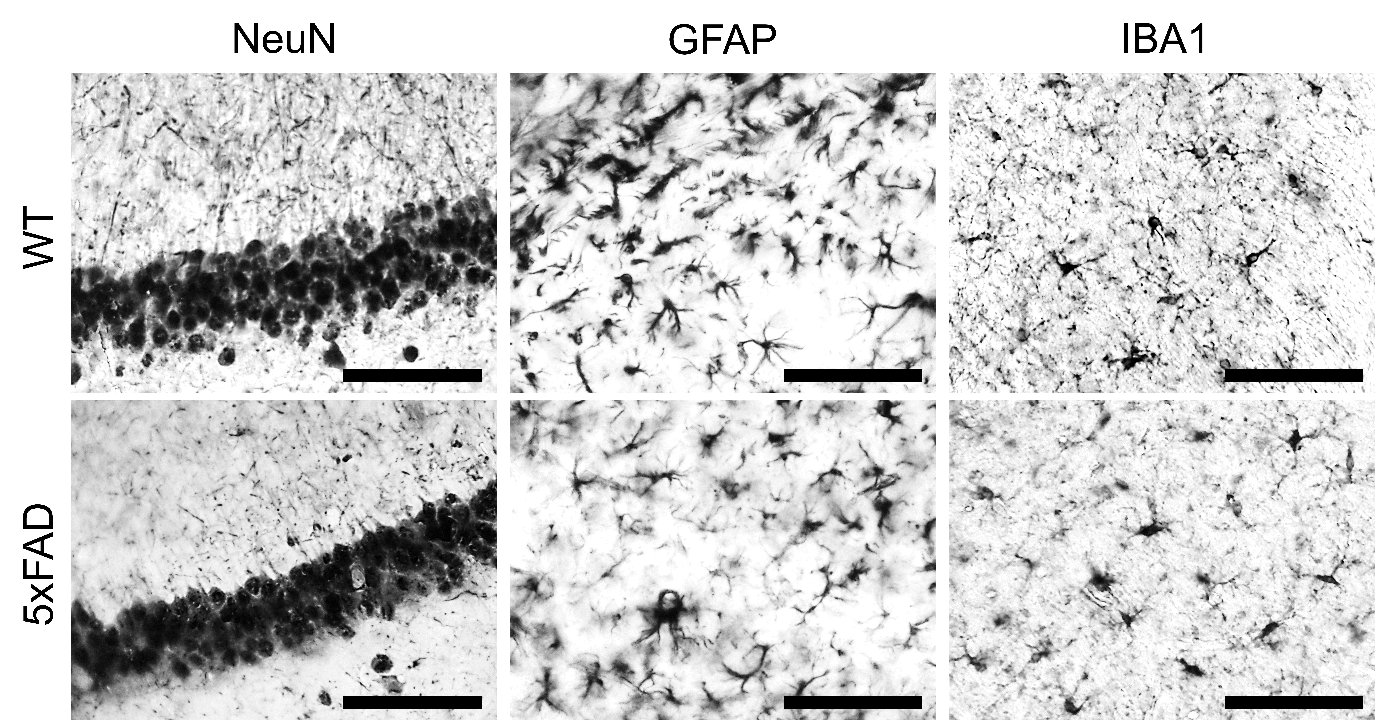


**Figure S8. Confirmation of cell-type-specific protein biotinylation on the brain tissue sections from WT and 5xFAD mice by chromogen staining.**

For the cell-type-specific biotinylation of mouse brain sections from WT and 5xFAD, we conducted iCAB using anti-NeuN, anti-GFAP, and anti-IBA1 antibodies for neurons, astrocytes, and microglia, respectively. To confirm the cell-type-specific protein biotinylation of the mouse brain sections, the biotinylation was detected using an ABC kit consisting of streptavidin and biotinylated HRP. Staining was done with Deep Space Black chromogen, which imparts a dark color on the biotinylated regions. These images were taken from the hippocampus region. The scale bar represents 100 µm.

**
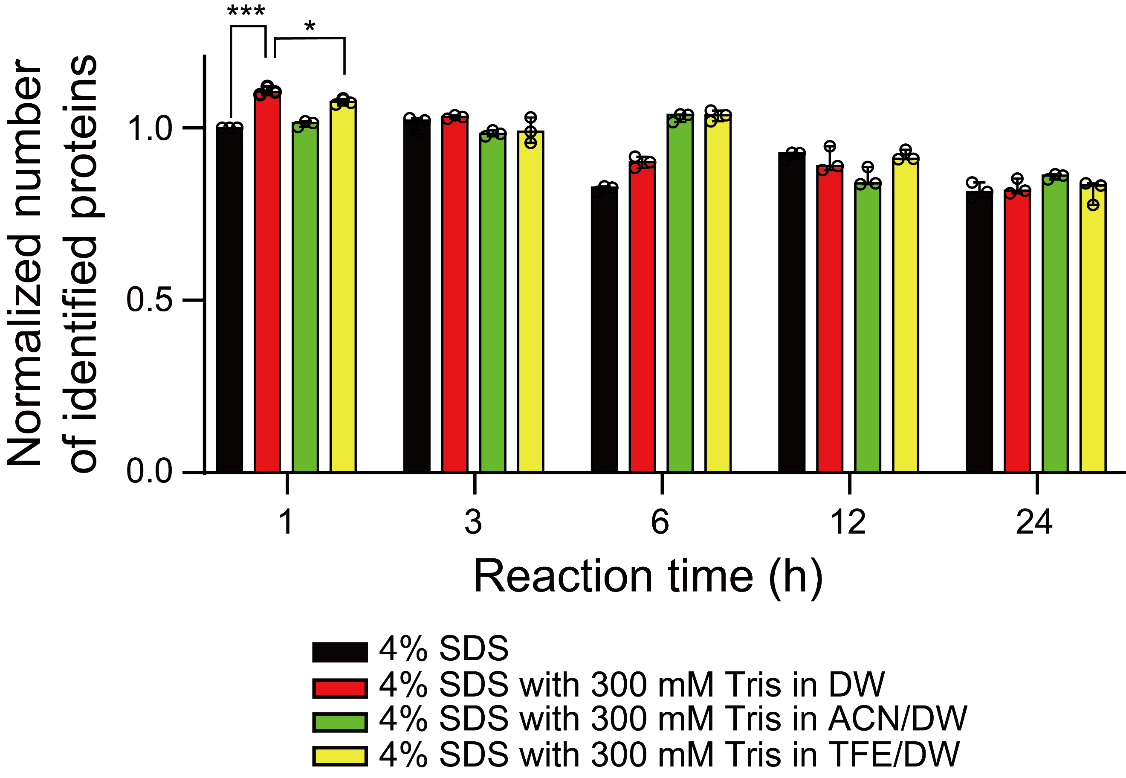
**

**Figure S9. Optimization of decrosslinking conditions for PFA-modified proteins from PFA-fixed mouse brain tissue sections.**

Various types of decrosslinking buffers were evaluated, including (1) 4% SDS, 1% SDC, and 50 mM of TEAB (black bar); (2) 4% SDS, 300 mM Tris, 1% SDC, and 50 mM of TEAB (red bar); (3) 4% SDS, 300 mM Tris, 1% SDC, 25 mM TEAB, and 50% ACN (green bar); (4) 4% SDS, 300 mM Tris, 1% SDC, 25 mM TEAB, and 50% TFE (yellow bar). Subsequently, PFA-fixed mouse brain tissue sections (n=3, technical replicates for each group) treated with these buffers were subjected to heating at 99°C for various reaction times: 1, 3, 6, 12, and 24 hours. The error bars indicate the standard deviation. An unpaired Student’s t-test was used for the statistical analysis (*: *p* < 0.05, ***: *p* < 0.001, and ns: not significant). Data are presented as mean ± standard deviation (SD).


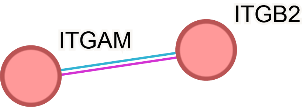


**Figure S10. STRING PPI analysis of the proteins from the most enriched WIKI Pathway of the bulk proteome.**

This STRING PPI analysis result contains 2 nodes with 1 edge. Experimental and database evidence were used for the active interaction source with 0.9 of a minimum required interaction score (average node degree: 0.182, average local clustering coefficient: 0.182, and PPI enrichment *P* value < 0.0616). Nodes for upregulated proteins are colored in red, while those for down-regulated proteins are colored in blue. The disconnected nodes have been removed from the network. Edges with purple and blue colors represent experimental and database evidence, respectively.

**
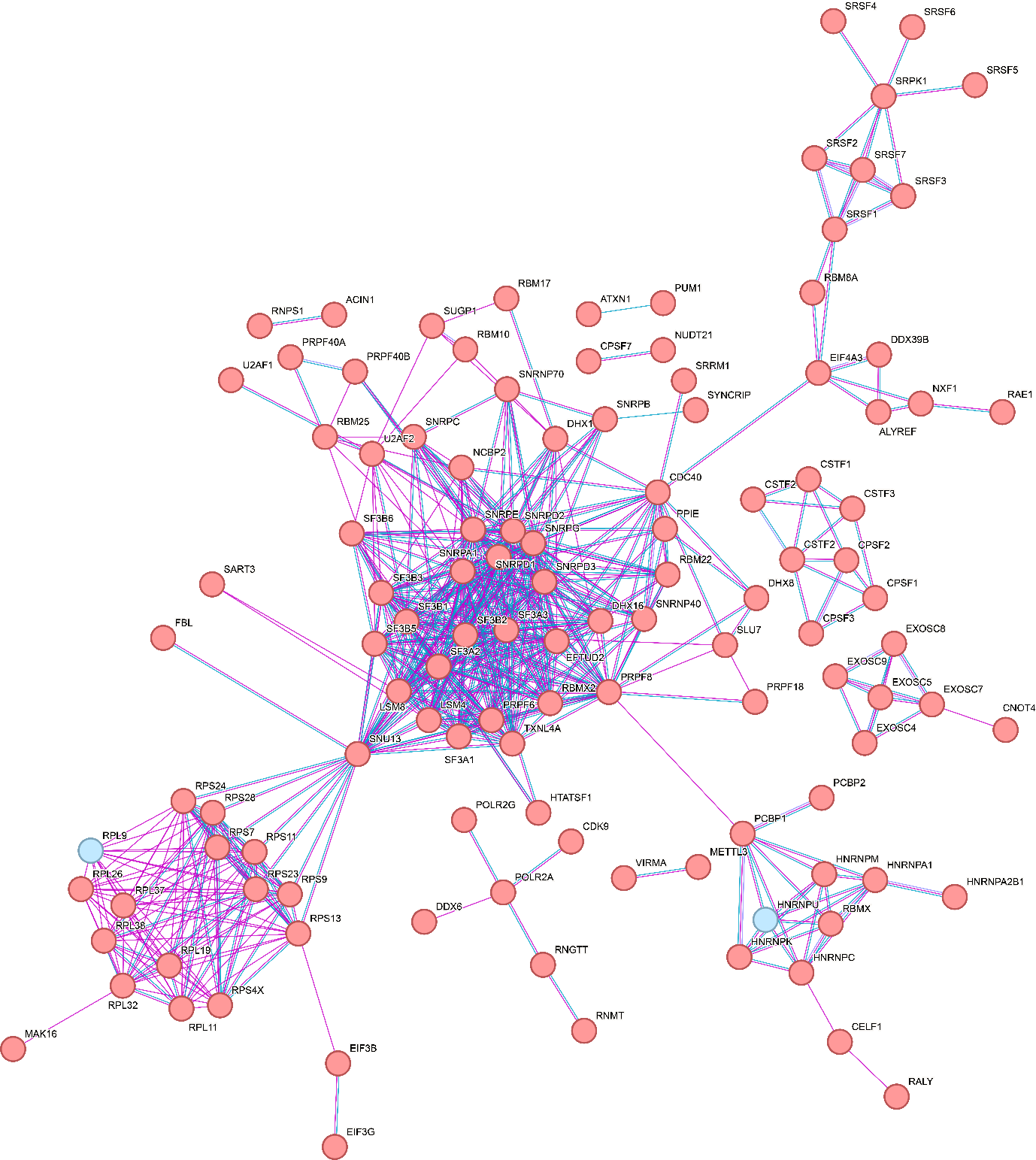
**

**Figure S11. STRING PPI analysis of the proteins from the most enriched WIKI Pathway of neuron proteome.**

This STRING PPI analysis result contains 118 nodes with 554 edges. Experimental and database evidence were used for the active interaction source with 0.9 of a minimum required interaction score (average node degree: 5.01, average local clustering coefficient: 0.575, and PPI enrichment *P* value < 1.0e^-16^). Nodes for up-regulated proteins are colored in red, while those for down-regulated proteins are colored in blue. The disconnected nodes have been removed from the network. Edges with purple and blue colors represent experimental and database evidence, respectively.

**
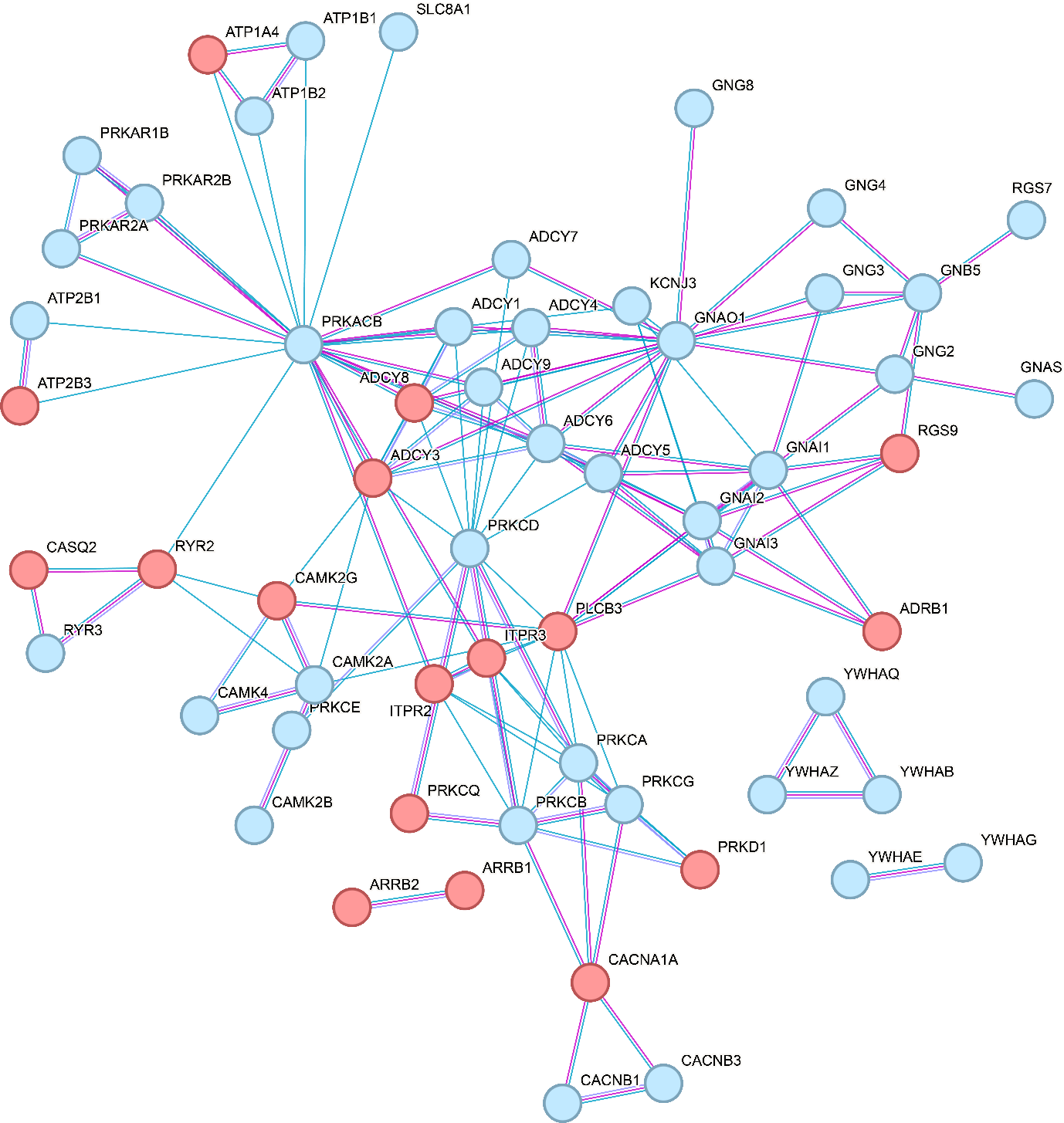
**

**Figure S12. STRING PPI analysis of the proteins from the most enriched WIKI Pathway of the astrocyte proteome.**

This STRING PPI analysis result contains 59 nodes with 138 edges. Experimental and database evidence were used for the active interaction source with 0.9 of a minimum required interaction score (average node degree: 3.78, average local clustering coefficient: 0.575, and PPI enrichment *P* value < 1.69e^-16^). Nodes for up-regulated proteins are colored in red, while those for down-regulated proteins are colored in blue. The disconnected nodes have been removed from the network. Edges with purple and blue colors represent experimental and database evidence, respectively.

**
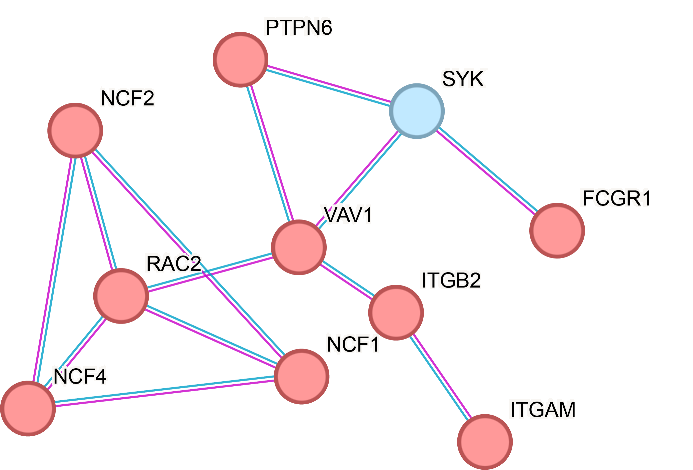
**

**Figure S13. STRING PPI analysis of the proteins from the most enriched WIKI Pathway of microglia proteome.**

This STRING PPI analysis result contains 10 nodes with 13 edges. Experimental and database evidence were used for the active interaction source with 0.9 of a minimum required interaction score (average node degree: 2, average local clustering coefficient: 0.538, and PPI enrichment *P* value < 1.0e^-12^). Nodes for up-regulated proteins are colored in red, while those for down-regulated proteins are colored in blue. The disconnected nodes have been removed from the network. Edges with purple and blue colors represent experimental and database evidence, respectively.


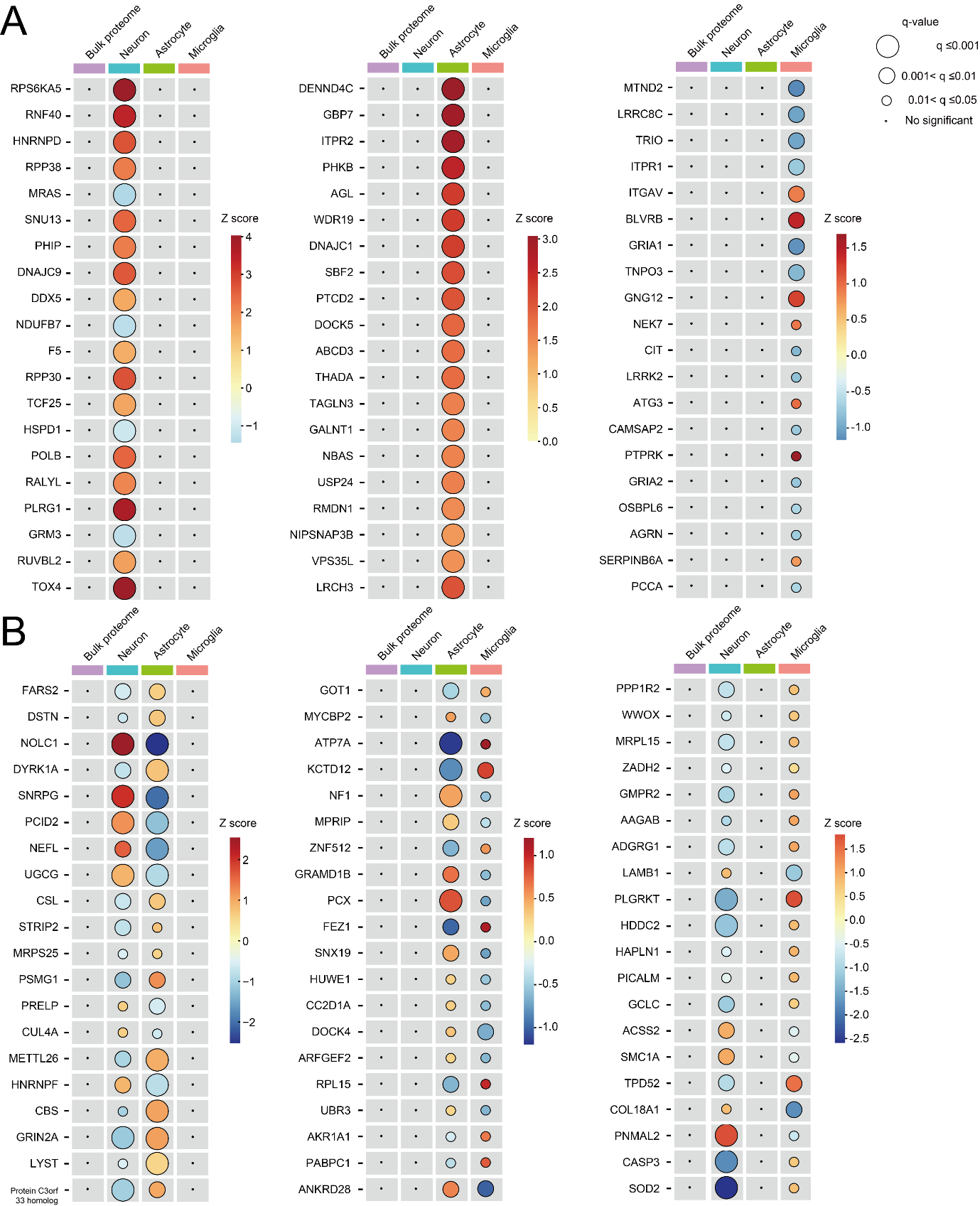


**Figure S14. List of proteins differentially expressed only in one cell type or in two cell types in opposite directions.**

A list of the top 20 proteins that are differentially expressed only in one cell type of 5xFAD mouse brain (A). The top 20 proteins are selected based on q-value.

A list of the top 20 proteins that were expressed in two different cell types in opposite directions (B). The top 20 proteins were selected as follows. All the proteins expressed in opposite directions in two different cell types were selected first, followed by adding Z-scores from the two different cell types. Then, the top 20 proteins with the smallest z-scores were selected.

**
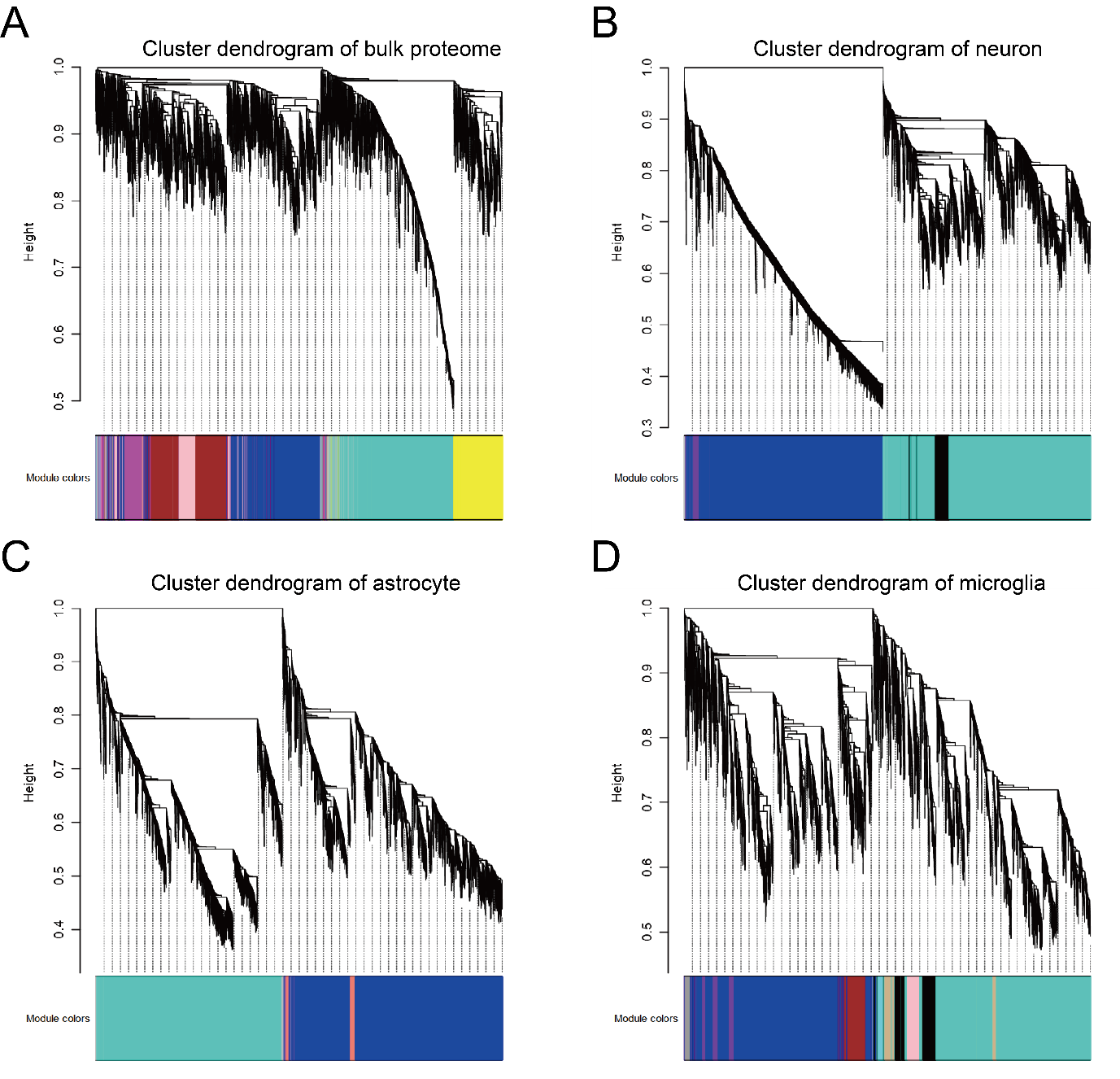
**

**Figure S15. The clustering dendrogram for WGCNA modules.**

The cluster dendrogram shows module clustering for the bulk proteome (A), neurons (B), astrocytes (C), and microglia (D). Various modules are distinguished by different colors. The grey bars depict proteins that are not affiliated with any other modules without exhibiting co-expression.
